# Optimal power load ranges for the power clean and hang power clean weight‐lifting derivatives: A systematic review and meta‐analysis

**DOI:** 10.14814/phy2.71154

**Published:** 2026-11-01

**Authors:** Jiaxuan Sun, Yiming Wu, Yifan Zhang, Lijun Shi, Li Li

**Affiliations:** ^1^ Department of Exercise Physiology Beijing Sport University Beijing China; ^2^ Laboratory of Sports Stress and Adaptation of General Administration of Sport Beijing Sport University Beijing China; ^3^ Shaanxi Normal University Shaanxi China

**Keywords:** hang power clean, optimal load, peak power, power clean

## Abstract

The aim of this study was to systematically evaluate the impact of different loads on the output power of the power clean (PC) and hang power clean (HPC). A systematic search of PubMed, Web of Science, EMBASE, and EBSCO identified 14 eligible studies involving 197 participants. The results of the meta‐analysis showed that compared to low loads (≤40% one‐repetition maximum [1RM]), moderate‐to‐high (>40% to ≤70% 1RM), high (>70% to ≤85% 1RM), and very high loads (>85% 1RM) produced greater acute peak power in both the PC (SMD = 0.58, 95% CI [0.17, 0.98]; SMD = 0.80, 95% CI [0.39, 1.21]; SMD = 0.68, 95% CI [0.16, 1.20] respectively) and the HPC (SMD = 0.58, 95% CI [0.28, 0.88]; SMD = 0.80, 95% CI [0.20, 1.40]; SMD = 0.94, 95% CI [0.55, 1.32] respectively) (all *p* < 0.01). In contrast, pairwise differences among Zones 2–4 were small to trivial and statistically nonsignificant for both the PC (SMD = 0.23, 0.19, and 0.06, respectively) and the HPC (SMD = 0.18, 0.01, and −0.21, respectively), consistent with a plateau in the power–load relationship once loads exceed 40% 1RM. Thus, the present evidence supports loads >40% 1RM as a broad range for high acute peak power expression. Pairwise comparisons among the >40% to ≤70%, >70% to ≤85%, and >85% 1RM zones did not demonstrate statistical superiority of any single higher‐load range.

## 1 INTRODUCTION

Developing muscular power is central to athletic performance, particularly in sports such as track and field and numerous team sports (e.g., basketball and soccer), where jumping and sprinting are constant requirements (Douglas et al., 2021; Redman et al., 2024). Higher muscular power (e.g., greater neuromuscular activation) has been linked with better competitive outcomes (Cronin & Hansen, 2005; Loturco et al., 2014). Explosive strength and peak power are closely related aspects of neuromuscular performance, as both depend on the ability to generate high levels of force rapidly. Consequently, peak power is commonly used as a practical indicator of explosive performance (Hinks et al., 2021). Training at or near loads that maximize mechanical power output has been proposed as an effective strategy for improving power‐related performance (Cormie, McCaulley, & McBride, 2007; Hoffman et al., 2005; McBride et al., 2002; Toji & Kaneko, 2004; Wilson et al., 1993). However, some researchers have reported that the load eliciting maximal acute power should not necessarily be regarded as a uniquely superior training load, as comparable improvements in power may be achieved across a range of surrounding loads or through strength‐oriented resistance training (Andersen et al., 2022; Gil‐Cabrera et al., 2021). Accordingly, the acute optimal power load may be better conceptualized as a reference point within a broader high‐power loading range rather than as a single mandatory training intensity. In applied practice, identifying the relative loads associated with high acute peak‐power output can nevertheless provide useful information for exercise testing and power‐oriented load prescription.

Weight‐lifting‐derived movements are widely recognized as effective tools for developing power, primarily due to their unique biomechanical properties that enable more effective triggering of a triple extension. Evidence indicates that training based on weight‐lifting enhances neuromuscular qualities that support explosive performance, including maximal strength, rate of force development, and technical skill (Hori et al., 2005; Waller et al., 2007). Researchers have also reported improvements in vertical jump performance following weight‐lifting training (Canavan et al., 1996; MacKenzie et al., 2014). That said, full weight‐lifting lifts (e.g., clean, jerk, snatch) require significant technical skill, mobility, and training experience, which may limit their applicability to novice or general athletic populations (Suchomel et al., 2015).

For this reason, many strength and conditioning programs incorporate weight‐lifting derivatives such as the power clean (PC) and hang power clean (HPC) (Kelly et al., 2014). The pulling sequence of the PC begins with the barbell on the floor and comprises the first pull, transition phase (also referred to as the scoop or double knee bend), and second pull, during which explosive triple extension of the hip, knee, and ankle occurs (Comfort et al., 2011a). In contrast, the HPC begins from a hang position and therefore omits the first pull (Caldwell, 2015). Both of them maintain the transition and second pull (i.e., triple extension), which drives power development (Comfort et al., 2012). However, compared with the jerk or snatch and their derivatives, they reduce the overall technical complexity and eliminates the overhead catch phase. Therefore, these derivatives are often more feasible. Given the common usage of PC and HPC, this review focuses on clarifying their optimal power loads for developing peak power.

Early work placed the optimal load for maximizing power in the PC and HPC somewhere between 70% and 100% 1RM (Soriano et al., 2015). However, as research on weight‐lifting derivatives has become more targeted, the previously established range has proven too broad to guide precise prescription (Soriano et al., 2015; Suchomel & Sole, 2017; Takei et al., 2021). A quantitative synthesis is therefore necessary to refine the optimal range and provide clearer, more actionable guidance for practitioners.

Accordingly, this meta‐analysis compared peak power in the PC and HPC across relative load ranges. By integrating results across studies, the aim was to identify the load range most favorable for maximal acute peak‐power expression and to support more practical power‐oriented load selection. Provides more accurate guidance for the development of personalized exercise programs by the general public and fitness trainers.

## 2 METHODS

This meta‐analysis was designed and reported in accordance with the Preferred Reporting Items for Systematic Reviews and Meta‐Analyses (PRISMA) guidelines (Page et al., 2021), ensuring transparency, reproducibility, and methodological rigor. The full PRISMA checklist is documented in S1 of the Electronic Supplementary Material [ESM].

### 2.1 Literature search strategy

Two investigators independently conducted systematic searches of PubMed, Web of Science, EBSCO, and EMBASE from database inception to December 14, 2025. No restrictions were placed on publication year or participant sex.

Search terms combined subject headings and free‐text terms, including: “power training,” “power clean,” “hang power clean,” “mechanical power,” “maximum power,” “power production,” “power development,” “peak power,” “optimal load,” and “power–load curve.” Reference lists of included studies were manually screened to identify additional relevant articles. The full search strategy, including search terms, is documented in S2 of the Electronic Supplementary Material [ESM].

### 2.2 Inclusion and exclusion criteria

Eligible studies met the following criteria: healthy participants with experience in the PC or HPC; experimental assessment of peak power under different relative loads; randomized or repeated‐measures designs; and peak power reported in watts (W).

Inclusion criteria:

1. Absolute power data reported (not normalized solely to body mass), presented in text or figures;
2. Loads prescribed relative to 1RM;
3. Peak power (W) is the primary outcome, measured via force platforms and incorporating body weight into calculations. This standard enhances consistency in power measurements across different studies and focuses the analysis on systemic power rather than barbell power (Dugan et al., 2004; Harris et al., 2010; Hori et al., 2008).

Exclusion criteria:

1. Reviews, meta‐analyses, or case reports;
2. Incomplete outcome data or unavailable full texts;
3. Load prescription not based on 1RM;
4. Participants with known injuries or special populations.

### 2.3 Study selection

All records were imported into EndNote 20.3 for duplicate removal. Two investigators independently screened titles and abstracts, followed by full‐text review of potentially eligible studies. Disagreements were resolved through discussion or adjudication by a third investigator. The selection process is illustrated using a PRISMA flow diagram.

### 2.4 Data extraction

Two investigators independently extracted data using a standardized form, including author, year, participant characteristics, exercise type (PC or HPC), relative loads (% 1RM), and corresponding peak power values (mean ± SD). When numerical data were not reported, values were extracted from figures using WebPlotDigitizer (version 4.8). Authors were contacted when clarification was required; studies lacking essential data were excluded.

### 2.5 Methodological quality assessment

Because the included evidence primarily consisted of acute experimental and repeated‐measures studies examining peak‐power responses across different loads, methodological quality was assessed using the five‐domain scoring framework previously applied by Soriano et al. (2015) to studies examining the load–power relationship in lower‐body resistance exercises. This framework was selected because its domains directly address methodological features relevant to acute power–load testing and the reproducibility of these experiments.

The five domains comprised: (1) sample description, including participant characteristics, population classification, and training status; (2) procedure description, including testing procedures, instructions to maximize performance, ordering of experimental conditions, and familiarization; (3) intervention characteristics, including exercise technique, loading conditions, number of trials, and recovery between trials; (4) measurement system, data collection, and data analysis, including instrumentation, sampling frequency, power calculation procedures, inclusion of body mass, and data‐processing software; and (5) results reporting, including measures of central tendency and variability. These domains were considered particularly relevant because participant training status, testing procedures, exercise execution, recovery, and power‐measurement methodology may influence acute peak‐power estimates.

Each of the five domains was scored as 1 when the methodological criterion was adequately met/reported and as 0 when the criterion was not met or when insufficient information was available to determine whether it was met, resulting in a total score ranging from 0 to 5. The total score was used as a descriptive summary of methodological reporting rather than as a substitute for consideration of individual methodological domains. The complete scoring criteria are shown in Figure 1, and domain‐level ratings for each of the 14 included studies are provided in S3 of the Electronic Supplementary Material [ESM]. Methodological quality scores were not used to exclude studies or to modify the statistical weights assigned to individual studies in the meta‐analysis.

**FIGURE 1:**
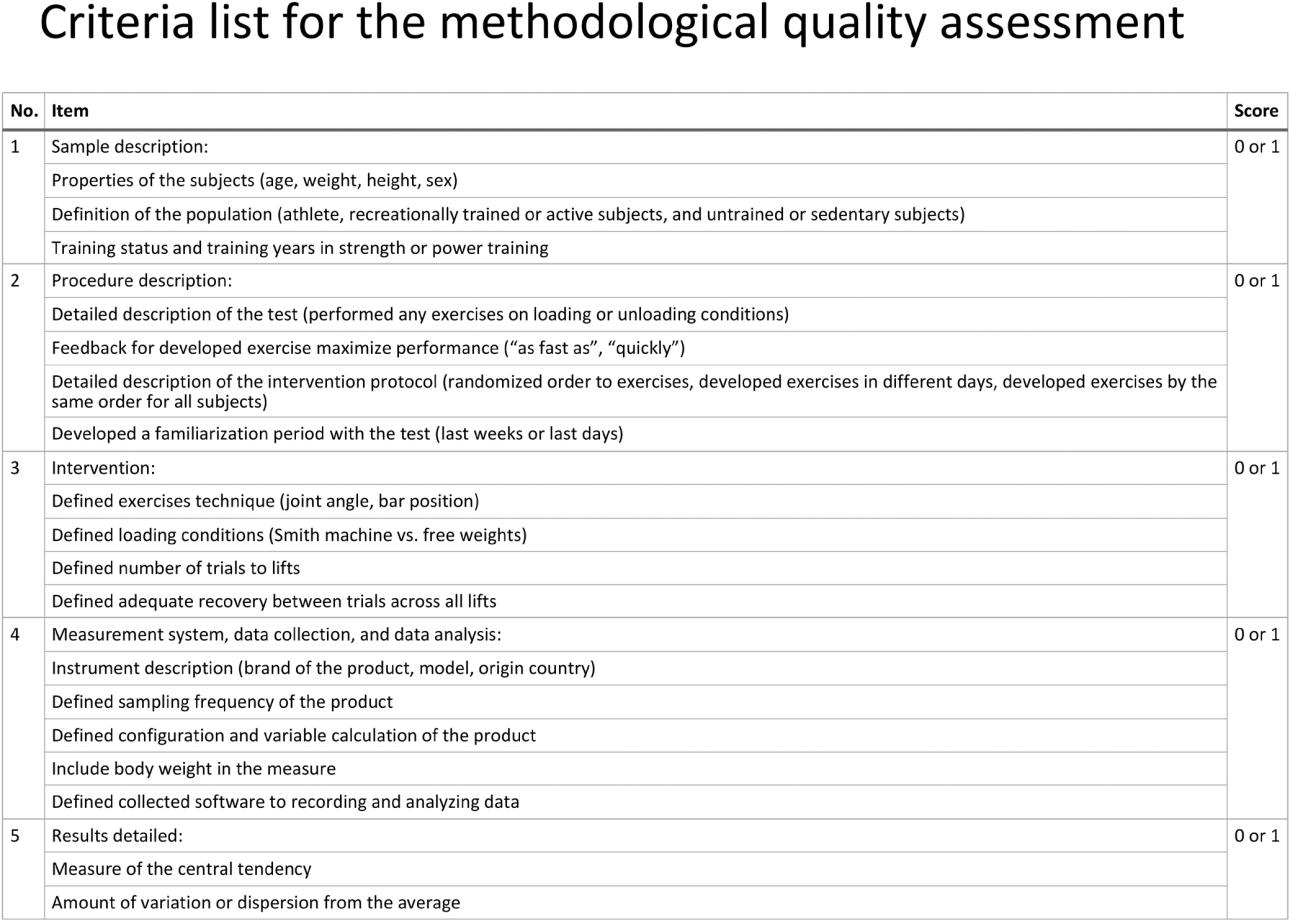
Criteria list for methodological quality assessment.

### 2.6 Statistical analysis

Meta‐analyses were conducted using RevMan 5.4.0 and Stata/MP 16.0. Standardized mean differences (Hedges' *g*) with 95% confidence intervals (CIs) were calculated to standardize effect estimates across studies and reduce the influence of differences in absolute power magnitude. Heterogeneity was assessed using Cochran's *Q* and *I* ^2^ statistics. Fixed‐effects models were used when *I* ^2^ ≤ 50%, and random‐effects models when *I* ^2^ > 50%. Sensitivity and subgroup analyses were performed for outcomes with high heterogeneity. Publication bias was assessed using Egger's test, with trim‐and‐fill applied when bias was detected.

### 2.7 Definition of load ranges

To mitigate statistical dependence resulting from multiple load conditions reported within the same study, a single observation was retained for each predefined relative load zone. Loads were categorized into four mutually exclusive relative‐load zones: ≤40% 1RM (Zone 1), >40% to ≤70% 1RM (Zone 2), >70% to ≤85% 1RM (Zone 3), and >85% 1RM (Zone 4). Representative target loads of 40%, 60%, 80%, and 90% 1RM were selected for Zones 1–4, respectively. When the exact representative load was reported, the corresponding peak‐power value was extracted. When the prespecified representative load was unavailable, the load associated with the highest observed peak power within the corresponding zone was retained. This selection rule was adopted because the primary objective of the present review was to identify the loading range most favorable for eliciting maximal acute peak power, rather than to estimate the mean power response across all loads within each interval. To avoid overrepresentation of studies reporting multiple load conditions within the same predefined zone, only one representative observation was retained for each load zone within each study. Loads were categorized into four mutually exclusive relative‐load zones: ≤40% 1RM (Zone 1), >40% to ≤70% 1RM (Zone 2), >70% to ≤85% 1RM (Zone 3), and >85% 1RM (Zone 4). Representative target loads of 40%, 60%, 80%, and 90% 1RM were selected for Zones 1–4, respectively. When the exact representative load was reported, the corresponding peak‐power value was extracted. When the prespecified representative load was unavailable, the load associated with the highest observed peak power within the corresponding zone was retained. For each pairwise comparison between load zones, each study contributed no more than one study‐level effect‐size estimate. A study reporting data for three or more load zones could therefore contribute to more than one pairwise comparison; however, each load‐zone contrast was analyzed as a separate meta‐analysis. Thus, effect‐size estimates arising from different load‐zone contrasts within the same study were not simultaneously entered into the same pooled analysis. Furthermore, the extracted values should be interpreted as representing the maximal observed response within each zone rather than the typical response across the entire loading interval.

## 3 RESULTS

### 3.1 Study selection

A total of 808 records were identified through the initial database search. After removing 474 duplicates and 34 irrelevant records, 266 studies were excluded based on title and abstract screening. Full‐text review was conducted for 34 studies, of which 20 were excluded for the following reasons: duplicate content (*n* = 2), unavailable full text (*n* = 3), incomplete outcome data (*n* = 12), and failure to prescribe loads based on 1RM (*n* = 3). Ultimately, 14 studies were included in the meta‐analysis (Comfort et al., 2012; Cormie, McBride, & McCaulley, 2007; Cormie, McCaulley, Triplett, et al., 2007; Hayashi et al., 2021; Kawamori et al., 2005; Kilduff et al., 2007; McBride et al., 2011; Mehls et al., 2022; Pennington et al., 2010; Suchomel, Beckham, et al., 2014; Suchomel, Wright, et al., 2014; Takei et al., 2021; Winchester et al., 2005; Xie et al., 2025). The study selection process is illustrated in Figure 2.

**FIGURE 2:**
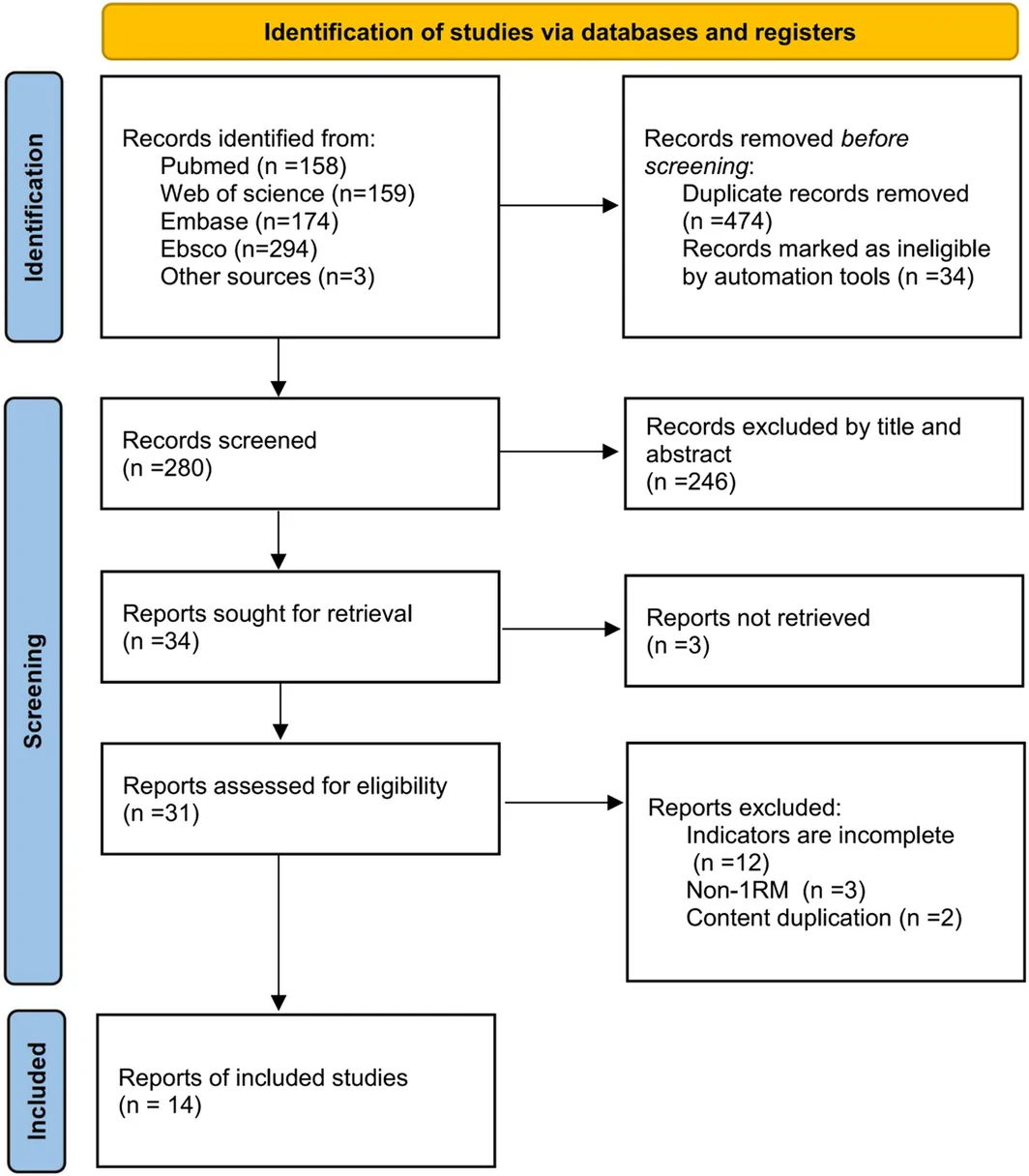
PRISMA flowchart of the study selection process.

### 3.2 Characteristics of included studies

Fourteen studies published between 2005 and 2025 were included, involving healthy participants aged 16–29 years, all of whom had experience performing weight‐lifting derivatives. A total of 197 participants were included; sex was not specified for 8 participants, while the remaining 189 participants were male. Of these, 121 participants performed the HPC, and 76 performed the PC. An overview of these studies is given in S4 of the Electronic Supplementary Material [ESM]. The methodological quality of each included study was assessed across the five predefined domains, and the study‐specific domain ratings and descriptive total scores are presented in S3 of the Electronic Supplementary Material [ESM].

### 3.3 Main meta‐analysis results

Zone 1 represents loads ≤40% 1RM, Zone 2 represents loads >40% to ≤70% 1RM, Zone 3 represents loads >70% to ≤85% 1RM, and Zone 4 represents loads >85% 1RM. Specific forest plots are shown in Figures 3 and 4.

**FIGURE 3:**
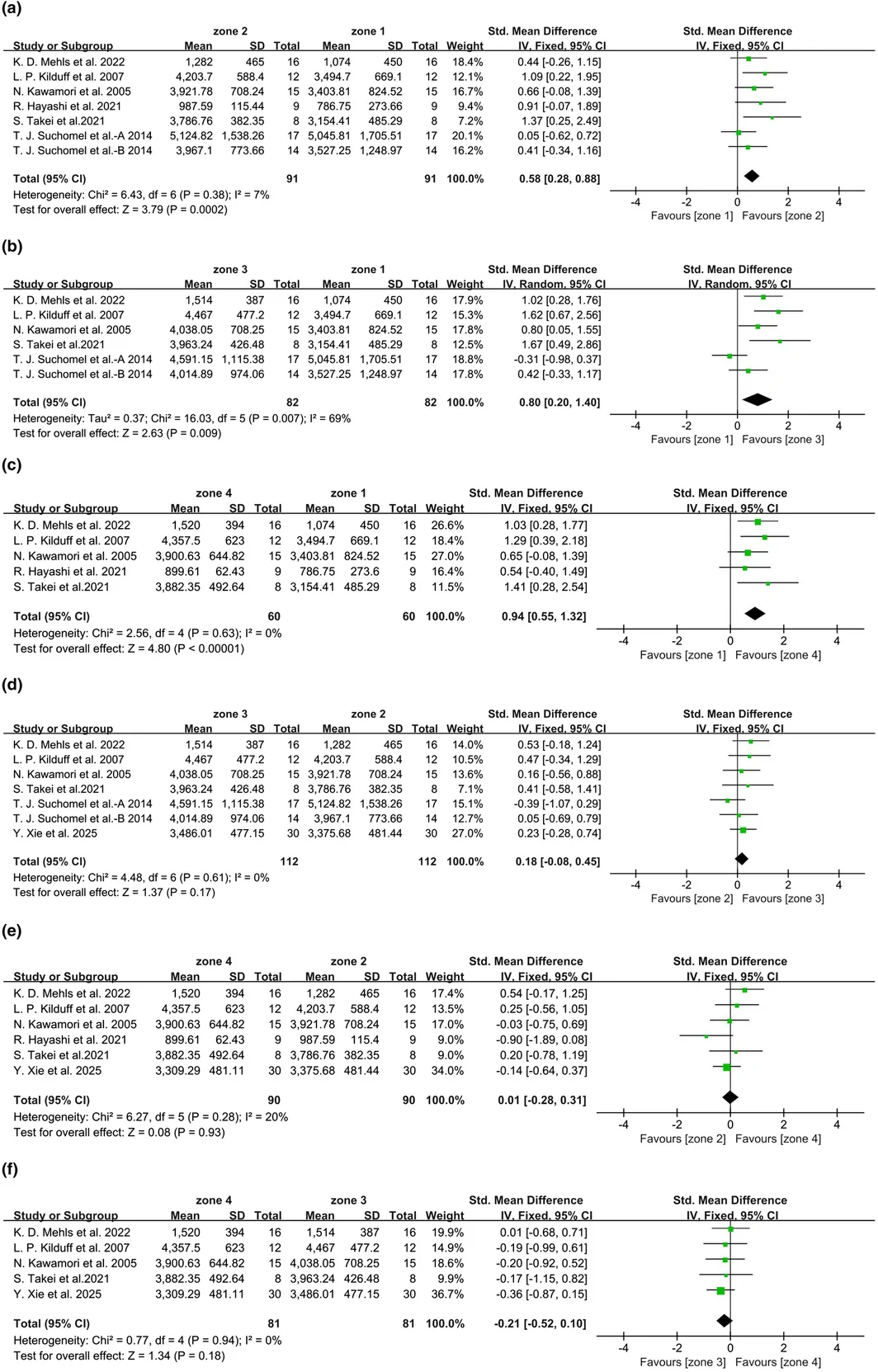
Forest plots of effect sizes for the hang power clean comparison. (a) Zone 1 (≤40% 1RM) with Zone 2 (>40% to ≤70% 1RM); (b) Zone 1 with Zone 3 (>70% to ≤85% 1RM); (c) Zone 1 with Zone 4 (>85% 1RM); (d) Zone 2 with Zone 3; (e) Zone 2 with Zone 4; and (f) Zone 3 with Zone 4. Effect sizes are presented as Hedges' *g* with 95% confidence intervals (CIs).

**FIGURE 4:**
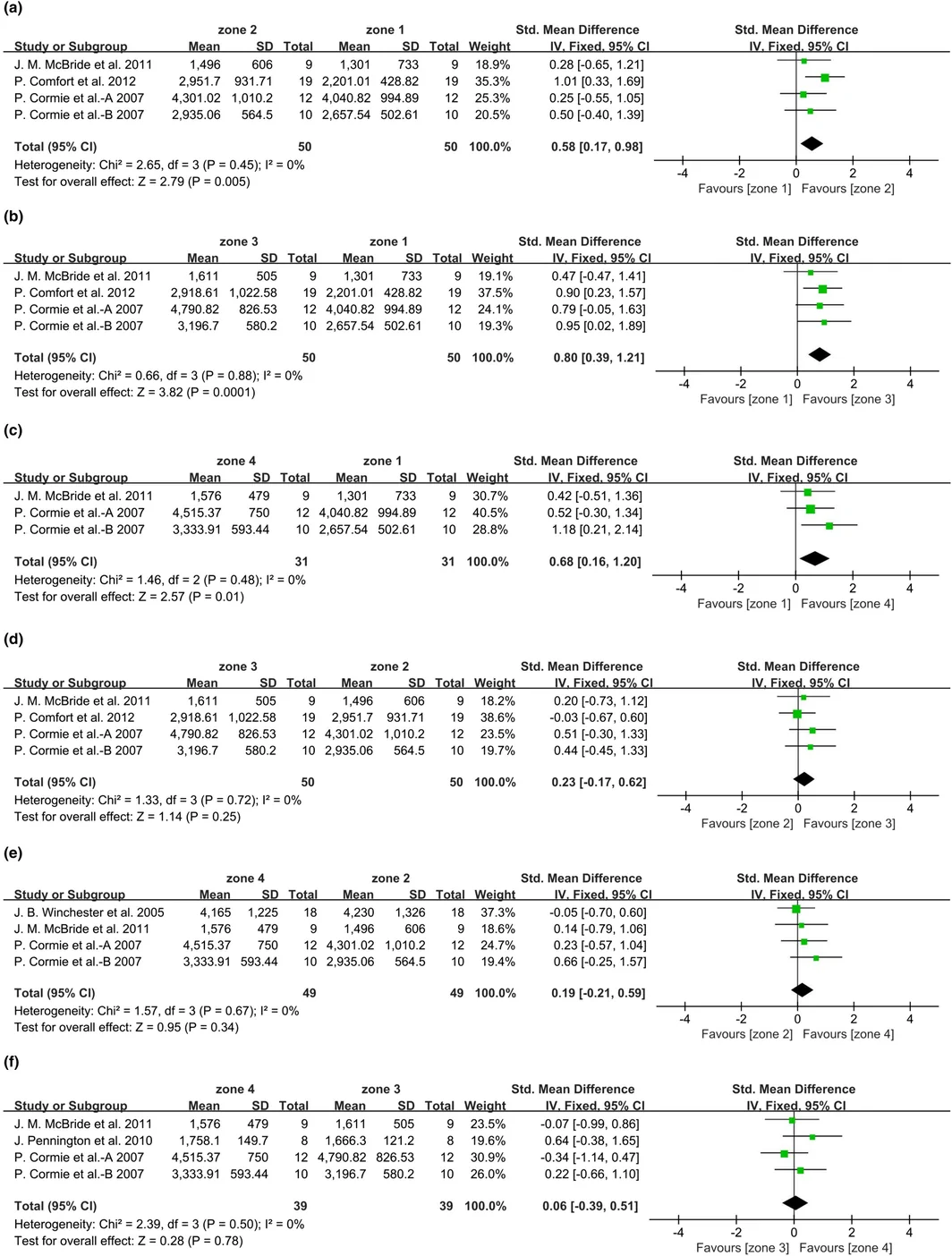
Forest plots of effect sizes for the power clean comparison. (a) Zone 1 (≤40% 1RM) with Zone 2 (>40% to ≤70% 1RM); (b) Zone 1 with Zone 3 (>70% to ≤85% 1RM); (c) Zone 1 with Zone 4 (>85% 1RM); (d) Zone 2 with Zone 3; (e) Zone 2 with Zone 4; and (f) Zone 3 with Zone 4. Effect sizes are presented as Hedges' *g* with 95% confidence intervals (CIs).

#### 3.3.1 Zone 1 versus Zone 2

For the HPC, loads in Zone 2 produced significantly greater peak power than those in Zone 1. Seven studies (Hayashi et al., 2021; Kawamori et al., 2005; Kilduff et al., 2007; Mehls et al., 2022; Suchomel, Beckham, et al., 2014; Suchomel, Wright, et al., 2014; Takei et al., 2021) were included in this comparison. The pooled analysis demonstrated a significant difference favoring Zone 2 (a, SMD = 0.58, 95% CI [0.28, 0.88], *p* < 0.001 in Figure 3), with low heterogeneity across studies (*I* ^2^ = 7%). However, Egger's test indicated a potential risk of publication bias (*p* = 0.009). This may be due to publication bias resulting from the inclusion of a limited number of articles; however, after handling with the trim‐and‐fill method, it was found that this publication bias had a limited impact on the conclusions.

Similarly, during the PC, peak power in Zone 2 was significantly greater than in Zone 1. Four studies (Comfort et al., 2012; Cormie, McBride, & McCaulley, 2007; Cormie, McCaulley, Triplett, et al., 2007; McBride et al., 2011) were included in this comparison. The results showed that Zone 2 loads elicited higher peak power (a, SMD = 0.58, 95% CI [0.17, 0.98], *p* = 0.005 in Figure 4), with no evidence of publication bias (*p* = 0.176) and high consistency among studies (*I* ^2^ = 0%).

#### 3.3.2 Zone 1 versus Zone 3

Compared with Zone 1, Zone 3 loads produced significantly greater peak power during the HPC. Six studies (Kawamori et al., 2005; Kilduff et al., 2007; Mehls et al., 2022; Suchomel, Beckham, et al., 2014; Suchomel, Wright, et al., 2014; Takei et al., 2021) were included in this comparison. The pooled results indicated a significant difference in favor of Zone 3 (b, SMD = 0.80, 95% CI [0.20, 1.40], *p* < 0.01 in Figure 3), although heterogeneity was substantial (*I* ^2^ = 69%). No significant publication bias was detected (*p* = 0.07).

Similarly, in the PC, peak power was significantly greater in Zone 3 than in Zone 1. Four studies (Comfort et al., 2012; Cormie, McBride, & McCaulley, 2007; Cormie, McCaulley, Triplett, et al., 2007; McBride et al., 2011) were included in this comparison, with the pooled estimate indicating an advantage for Zone 3 (b, SMD = 0.80, 95% CI [0.39, 1.21], *p* < 0.001 in Figure 4). No evidence of publication bias was observed (*p* = 0.517), and heterogeneity was negligible (*I* ^2^ = 0%).

#### 3.3.3 Zone 1 versus Zone 4

During the HPC, Zone 4 loads produced significantly greater peak power than Zone 1 loads. Five studies (Hayashi et al., 2021; Kawamori et al., 2005; Kilduff et al., 2007; Mehls et al., 2022; Takei et al., 2021) were included. The pooled analysis showed a significant difference favoring Zone 4 (c, SMD = 0.94, 95% CI [0.55, 1.32], *p* < 0.01 in Figure 3), with excellent consistency among studies (*I* ^2^ = 0%). No significant publication bias was detected (*p* = 0.465).

Likewise, in the PC, peak power in Zone 4 was significantly greater than in Zone 1. Three studies (Cormie, McBride, & McCaulley, 2007, Cormie, McCaulley, Triplett, et al., 2007, McBride et al., 2011) were included in this comparison, and the pooled analysis showed a significant difference (c, SMD = 0.68, 95% CI [0.16, 1.20], *p* = 0.01 in Figure 4), with no heterogeneity (*I* ^2^ = 0%) and no evidence of publication bias (*p* = 0.621).

#### 3.3.4 Zone 2 versus Zone 3

For the HPC, no significant difference in peak power was observed between Zone 2 and Zone 3. Seven studies (Kawamori et al., 2005; Kilduff et al., 2007; Mehls et al., 2022; Suchomel, Beckham, et al., 2014; Suchomel, Wright, et al., 2014; Takei et al., 2021; Xie et al., 2025) were included, and the pooled effect size indicated no significant difference (d, SMD = 0.18, 95% CI [−0.08, 0.45], *p* = 0.17 in Figure 3), with excellent consistency across studies (*I* ^2^ = 0%). Egger's test showed no evidence of publication bias (*p* = 0.677).

Similarly, for the PC, peak power did not differ significantly between Zone 2 and Zone 3. Four studies (Comfort et al., 2012, Cormie, McBride, & McCaulley, 2007, Cormie, McCaulley, Triplett, et al., 2007, McBride et al., 2011) were included in this comparison, and the pooled analysis showed a nonsignificant result (d, SMD = 0.23, 95% CI [−0.17, 0.63], *p* = 0.25 in Figure 4), with no heterogeneity (*I* ^2^ = 0%) and no publication bias detected (*p* = 0.268).

#### 3.3.5 Zone 2 versus Zone 4

In the HPC, no significant difference in peak power was observed between Zone 2 and Zone 4. Six studies (Hayashi et al., 2021; Kawamori et al., 2005; Kilduff et al., 2007; Mehls et al., 2022; Takei et al., 2021; Xie et al., 2025) were included, and the pooled effect size indicated no significant difference (e, SMD = 0.01, 95% CI [−0.28, 0.31], *p* = 0.93 in Figure 3). Heterogeneity was low (*I* ^2^ = 20%), and no publication bias was detected (*p* = 0.885).

Similarly, in the PC, no significant difference in peak power was found between Zone 2 and Zone 4. Four studies (Cormie, McBride, & McCaulley, 2007; Cormie, McCaulley, Triplett, et al., 2007; McBride et al., 2011; Winchester et al., 2005) were included in this comparison, and the pooled analysis showed no significant difference (e, SMD = 0.19, 95% CI [−0.21, 0.59], *p* = 0.34 in Figure 4), with excellent consistency across studies (*I* ^2^ = 0%) and no evidence of publication bias (*p* = 0.257).

#### 3.3.6 Zone 3 versus Zone 4

For the HPC, no significant difference in peak power was observed between Zone 3 and Zone 4. Five studies (Kawamori et al., 2005; Kilduff et al., 2007; Mehls et al., 2022; Takei et al., 2021; Xie et al., 2025), involving a total of 81 participants, were included in this comparison. Each study contributed one study‐level effect‐size estimate to this pairwise meta‐analysis. The pooled analysis showed no significant difference (f, SMD = −0.21, 95% CI [−0.52, 0.10], *p* = 0.18 in Figure 3), with good consistency among studies (*I* ^2^ = 0%) and no publication bias (*p* = 0.305).

Similarly, in the PC, peak power did not differ significantly between Zone 3 and Zone 4. Four studies (Cormie, McBride, & McCaulley, 2007; Cormie, McCaulley, Triplett, et al., 2007; McBride et al., 2011; Pennington et al., 2010) were included in this comparison, and the pooled analysis showed a nonsignificant result (f, SMD = 0.06, 95% CI [−0.39, 0.51], *p* = 0.78 in Figure 4), with no heterogeneity (*I* ^2^ = 0%) and no evidence of publication bias (*p* = 0.127).

### 3.4 Sensitivity analysis

The pooled effect sizes showed some sensitivity to individual studies; however, exclusion of any single study did not materially alter the direction or magnitude of the pooled effects (Figure 5). Funnel plots are provided in S5 of the Electronic Supplementary Material [ESM]. Subgroup analysis revealed no statistically significant subgroup effects (*p* = 0.07), suggesting that training status did not significantly modify the effect of Zone 2 compared with Zone 1. However, the limited number of studies included in subgroup analyses may have reduced the ability to detect subgroup differences. Specific subgroup analyses are presented in Figure 6.

**FIGURE 5:**
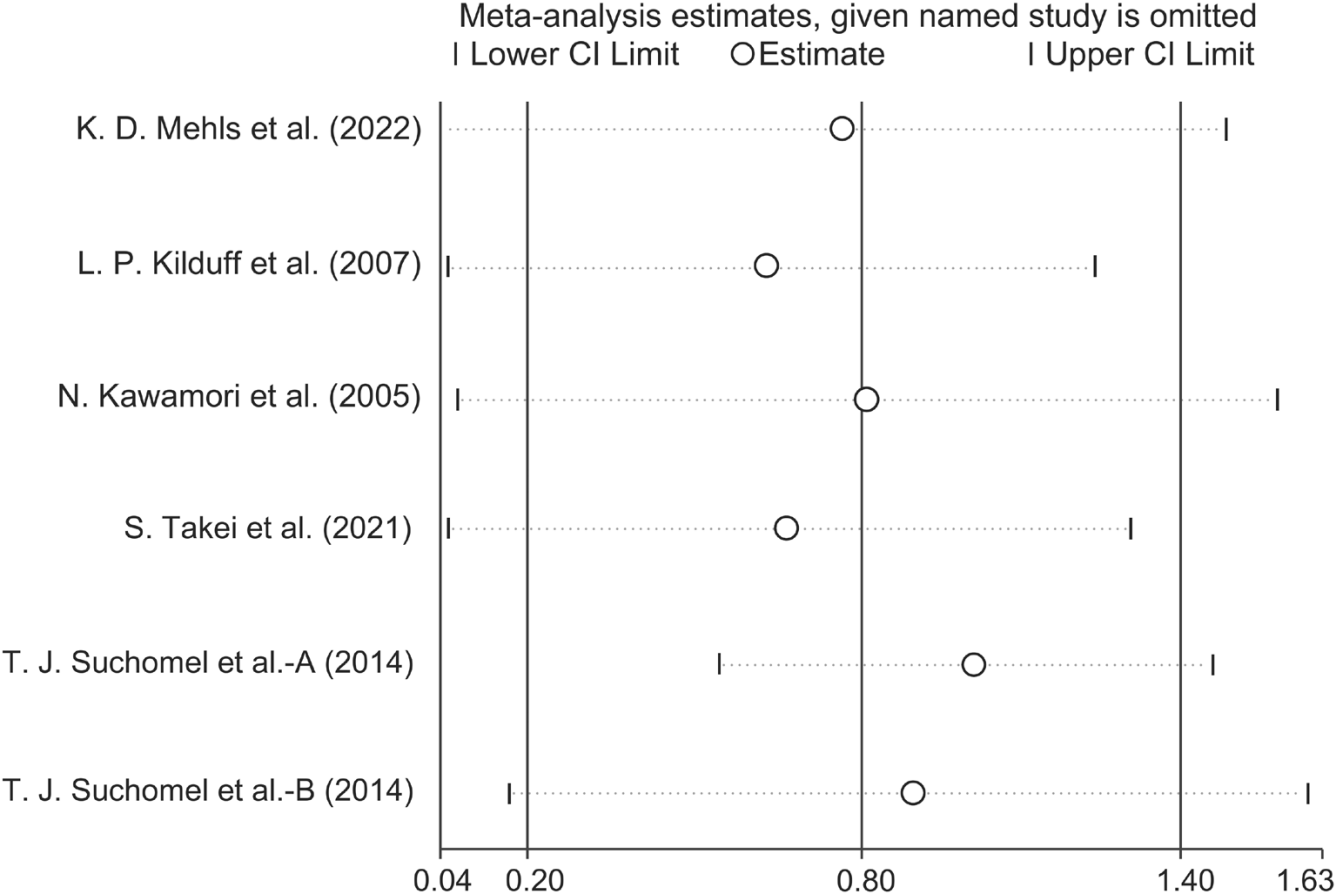
Sensitivity analysis performed on Zone 1 and Zone 3.

**FIGURE 6:**
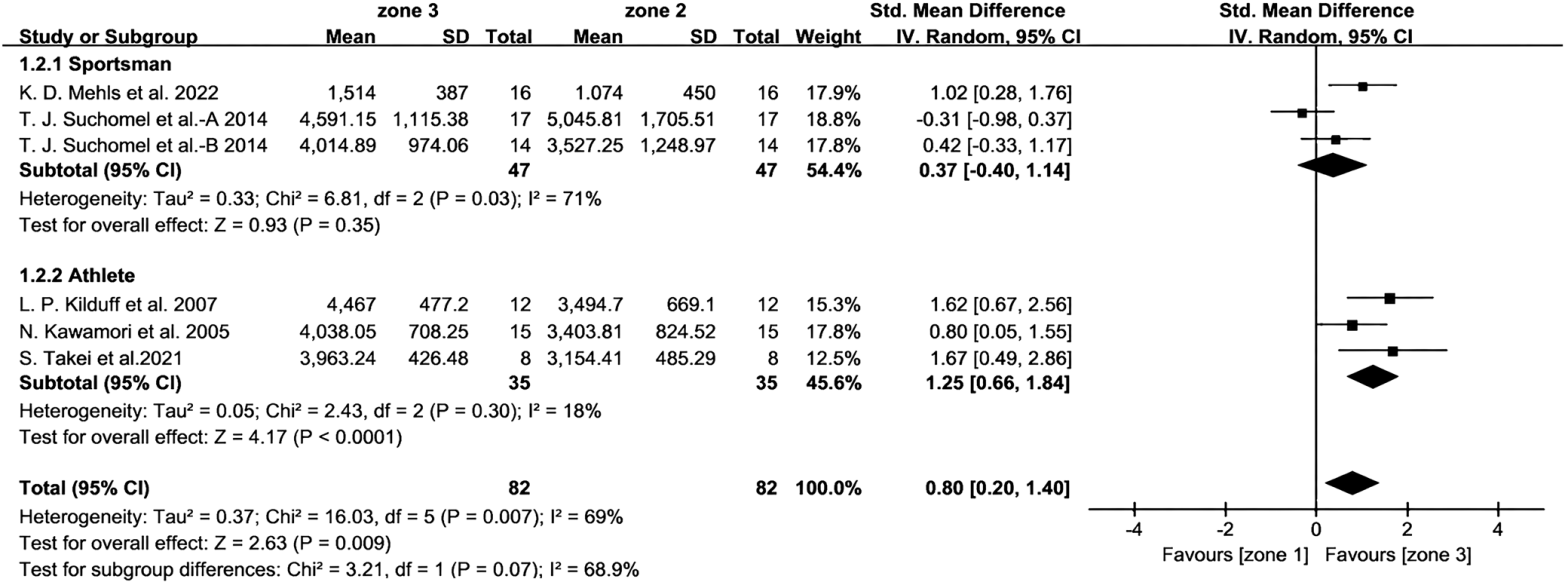
Subgroup analysis of Zone 1 and Zone 3. Subgroup analysis of Zone 1 and Zone 3 from the perspective of sports experience, where “athlete” refers to individuals with professional sports experience, and “recreationally trained” refers to enthusiasts who have not participated in formal competitions.

Notably, among recreationally trained, no significant difference in peak power was observed between Zone 1 and Zone 3 during the HPC, and heterogeneity remained high (*I* ^2^ = 71%). This may be attributable to one study (Suchomel, Wright, et al. 2014) that included both athletes and recreationally trained.

### 3.5 Publication bias

Publication bias detected in the comparison between Zone 1 and Zone 2 for the HPC was further examined using the trim‐and‐fill method. As shown in Figure 7: after adjustment, the pooled effect size remained significant (SMD = 0.44, 95% CI [0.18, 0.71]), indicating that publication bias had a limited impact on the robustness of the conclusion.

**FIGURE 7:**
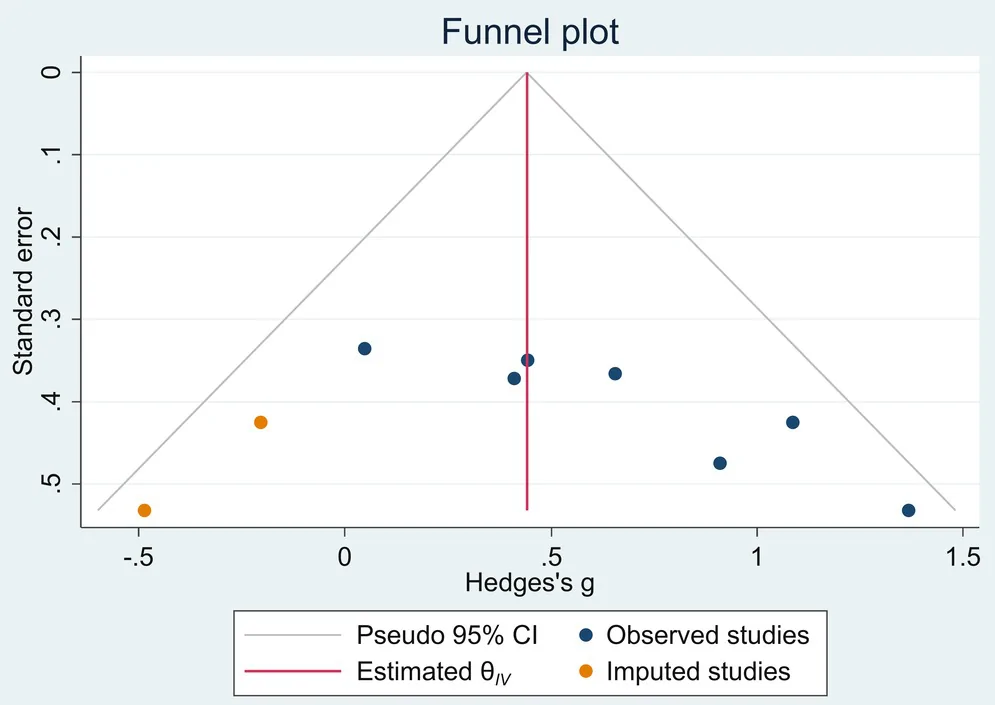
Analyzing publication bias using the trim‐and‐fill method.

## 4 DISCUSSION

We synthesized 14 studies published up to December 14, 2025, involving 197 participants, to compare peak power during the PC and HPC across relative load ranges. Overall, peak power increased markedly once loads exceeded 40% 1RM. Beyond that point, differences among the >40% to ≤70%, >70% to ≤85%, and >85% 1RM load ranges were generally small and nonsignificant, indicating a plateau in the power‐load relationship.

### 4.1 Nonlinear characteristics of peak power

For both the PC and HPC, peak power was numerically highest in Zone 4; however, the pairwise differences between Zones 3 and 4 were not statistically significant. Because pairwise differences among Zones 2, 3, and 4 were small and statistically nonsignificant, the present findings do not identify a single optimal loading zone above 40% 1RM. Instead, these results are consistent with a relatively broad high‐power range once loads exceed 40% 1RM. One practical consideration is noteworthy: Peak power was quantified using force plates in all included studies, and our eligibility criteria required body mass to be incorporated into the power calculation. The present findings are therefore most directly applicable to force‐plate‐derived system power rather than barbell‐only power. These measures should not be considered interchangeable because they represent different mechanical quantities. Some methodological variation may nevertheless remain across studies (Cormie, McBride, & McCaulley, 2007; Lake et al., 2012; McBride et al., 2011). Peak‐power estimates can be affected by how velocity is derived from ground reaction force data, the treatment of body weight and initial conditions, signal processing procedures, and the time window used to identify peak power. Although the use of Hedges' *g* reduces the influence of differences in absolute power scales, it cannot fully account for differences in the underlying computational procedures. The pooled results should therefore be interpreted primarily as relative differences in acute peak power between loading zones rather than as a universal absolute peak‐power reference. Subsequent research should further refine this method to further determine whether different computational approaches would yield different power results.

Across both lifts, the low‐load zone (≤40% 1RM) consistently yielded the lowest peak power. As loads exceeded 40% 1RM, peak power increased rapidly and then stabilized. Given that power represents the interaction of force and velocity, low loads may not provide sufficient external resistance to facilitate high force production, even when bar velocity is elevated (Martínez‐Cava et al., 2019), nor is the intention to pursue high power output evident under light load conditions (Suchomel et al., 2016). Furthermore, very light loads may prompt athletes to focus on controlling the bar path, which can diminish force application and ultimately reduce velocity and power. As loads increase into moderate‐to‐high ranges, force rises significantly while velocity remains relatively high, creating optimal conditions for maximal power development (Soriano et al., 2015). At very high loads (>85% 1RM), although the force generated by muscles increases, peak power may not further increase due to a reduction in movement speed and may even decrease in certain individuals (Bakhshinejad et al., 2025).

Collectively, the power–load relationship in the PC and HPC exhibits a pattern less resembling a straight line and more akin to a threshold followed by a plateau. In other words, the optimal load is better conceptualized as a relatively broad high‐power window rather than as a single precise point.

Biomechanical differences between the PC and HPC likely contribute to variations in power expression. The pulling sequence of the PC comprises the first pull, transition phase (scoop or double knee bend), and second pull, with explosive triple extension of the hip, knee, and ankle occurring during the second pull (Comfort et al., 2011a). Because the PC begins from the floor and includes the first pull, it involves a greater total barbell displacement than the HPC, which begins from the hang position and omits the first pull. Consequently, the overall barbell displacement of the PC is greater, and the time to reach peak power is longer, resulting in a lower stimulus for maximal explosive strength (Comfort et al., 2011b). The HPC eliminates the first pull phase and may place greater emphasis on rapid force production during the second pull and triple extension (Caldwell, 2015), and its shorter barbell displacement results in more focused force generation (Comfort et al., 2011b). These structural differences may contribute to different kinetic and kinematic demands between the two derivatives. The longer pulling sequence of the PC may permit a longer period of force application, whereas the shorter pulling sequence of the HPC may place relatively greater emphasis on rapid force production. These proposed mechanisms are based on biomechanical considerations and previous literature and should not be interpreted as outcomes directly demonstrated by the present meta‐analysis, which evaluated peak power rather than force–velocity profiles, impulse, or rate of force development. It may be possible to better utilize and exercise the force generation of the body during the stretch‐shortening cycle due to the process of lowering the barbell above the patella during the initiation of a HPC movement (Meechan et al., 2024). These biomechanical differences should be interpreted as practical considerations for movement selection based on training‐specific demands, rather than as evidence that a particular derivative form is inherently more suitable for a specific movement. Furthermore, these suggestions should not be taken as evidence of specific performance advantages or long‐term training adaptations.

### 4.2 Load selection in actual training

Although Zones 2, 3, and 4 produced broadly comparable peak power, these loading ranges should not necessarily be regarded as interchangeable in training practice because they impose different force–velocity profiles. Load selection should therefore be guided by the intended training emphasis. Loads of >40% to ≤70% 1RM can provide a high‐power stimulus while allowing relatively high movement velocity, maintaining technical quality, and facilitating fatigue management. In contrast, loads of >70% to ≤85% 1RM impose greater external‐load demands than the >40% to ≤70% range while still permitting high acute power output. However, the present meta‐analysis does not demonstrate that this range produces greater peak power than the adjacent higher load zones. At loads >85% 1RM, the additional force requirement is accompanied by a reduction in movement velocity, and the present results indicate no clear additional benefit for peak power compared with >70% to ≤85% 1RM. Importantly, however, peak power also did not differ significantly between the >40% to ≤70% and >70% to ≤85% 1RM ranges; therefore, the present findings do not establish >70% to ≤85% 1RM as a statistically superior loading zone. Thus, the optimal loading strategy should be interpreted in relation to the specific demands and objectives of the training program rather than as a single universally optimal load.

Although peak power at >70% to ≤85% 1RM was comparable to that observed at >85% 1RM, the present analysis identified no clear additional peak‐power benefit from increasing the load beyond 85% 1RM. Accordingly, >70% to ≤85% 1RM may be considered one practical option within the broader high‐power loading range, particularly when a greater force emphasis is desired. Heavier loads may still be incorporated when required by other training objectives, but their use cannot be justified on the basis of greater acute peak‐power output from the present findings. Overall, the present findings support a broad high‐power loading range above 40% 1RM rather than a single statistically superior zone. Although >70% to ≤85% 1RM may be considered for acute peak‐power assessment in some practical contexts, the pooled comparisons do not demonstrate that this range produces greater peak power than the adjacent higher‐load zones. In training contexts, moderate and heavier loads may be selected according to the intended force–velocity emphasis and broader training objectives. The biomechanical differences between the PC and HPC should also be considered when selecting the exercise derivative. In practical training, the biomechanical characteristics of the PC and HPC may be considered when selecting between derivatives according to the desired force–velocity emphasis of the training objective. However, because the present meta‐analysis evaluated acute peak power rather than sport‐specific performance or long‐term training adaptations, these considerations should not be interpreted as evidence that either derivative is superior for a particular sport or performance outcome.

It is also important to note that peak power is already relatively high when loads exceed 40% 1RM. Further increasing the load does not necessarily result in greater power output. It is also important to note that acute peak power was already relatively high once loads exceeded 40% 1RM, and further increases in load did not consistently produce greater peak power. Therefore, the present data support a broad high‐power loading range rather than a single load zone that can be considered optimal across individuals.

In practical applications, optimal power load is typically regarded as a reference point rather than as a strictly defined value. Coaches frequently use velocity‐based training (VBT) to track barbell velocity in real time, thereby inferring power output. This allows coaches to adjust loads on a daily basis and monitor fatigue (Weakley et al., 2021). Therefore, VBT provides practitioners with a practical framework to design and adjust training loads based on athletes' actual performance capabilities during specific training sessions (González‐Badillo & Sánchez‐Medina, 2010). In practice, this specifically means meaningful reductions in movement velocity may reflect an acute decline in neuromuscular readiness, especially following exposure to heavy loading or high training volumes. In consideration of practical circumstances, practitioners may consider modifying the prescribed load, reducing total volume, extending recovery intervals, or terminating a set before excessive fatigue compromises movement quality (Pareja‐Blanco et al., 2017; Sánchez‐Medina & González‐Badillo, 2011). However, velocity data should not be interpreted in isolation (Pérez‐Castilla et al., 2020). Bar path, technical consistency, and the athlete's ability to reproduce efficient movement patterns remain essential considerations, as high power output is most valuable when it is achieved through controlled, coordinated, and sport‐relevant mechanics (Cormie et al., 2011; Pérez‐Castilla et al., 2020). Integrating VBT may therefore assist coaches in individualizing power‐oriented training and managing fatigue (González‐Badillo & Sánchez‐Medina, 2010; Pareja‐Blanco et al., 2017; Sánchez‐Medina & González‐Badillo, 2011). However, load selection should also account for the technical and physical demands associated with heavier loading.

### 4.3 Limitations and evidence boundaries

Several limitations should be considered. While using broad load categories provides a practical means for comparing studies, this approach approximates a continuous power–load curve and may obscure finer individual differences in optimal load. Instead of performing weighted processing on the power corresponding to loads in different intervals, a specific value within the interval was selected, which may affect the results. Most participants were trained and experienced with weight‐lifting derivatives; their technical proficiency and relative strength may enable them to sustain favorable force‐velocity profiles at heavier loads, potentially shifting the optimal range upward (Stone et al., 2003). This may lead to the production of more pronounced evidence boundaries. These results tend to reflect “optimal load for acute power” rather than “optimal load for long‐term adaptations,” and whether this represents the optimal power load for long‐term training still requires further validation through more high‐quality long‐term intervention studies. Most participants were male; therefore, it remains uncertain whether the same plateau pattern applies to female athletes. The limited number of studies comparing PC loads may reduce the precision of the estimated optimal range. Additionally, because multiple loads were reported within the same zone in some studies, selecting the highest observed peak‐power value may have introduced a degree of selection bias, as zones with more available load conditions had greater opportunities to capture a higher response. Future studies using standardized load testing protocols across all zones are needed to minimize this limitation.

Publication bias was identified in the HPC comparison between Zone 1 and Zone 2. However, trim‐and‐fill analyses indicated that the primary conclusions remain robust. The high heterogeneity observed in certain comparisons may also reflect differences in participant characteristics, exercise execution, load distribution, and power‐processing procedures. Although force plates were used and body mass was incorporated into the power calculations in all included studies, methodological choices involved in deriving velocity and identifying peak power may still have contributed to between‐study variability. The use of Hedges' *g* reduces the influence of differences in absolute power scales, but it cannot fully account for variation in the underlying computational procedures. The pooled estimates should therefore be interpreted primarily as relative differences in acute peak power between loading zones rather than as universal absolute peak‐power reference values. This underscores the necessity for more standardized, high‐quality studies.

### 4.4 Future research directions

Future research should consider the following: (1) incorporate additional high‐quality studies to characterize the power–load relationship more precisely across loads >40% 1RM and to determine whether any narrower loading range consistently maximizes acute peak power; (2) when the loads differ by 10% 1RM, there is no significant difference in load power (Harris et al., 2010). Therefore, subsequent research should focus more on exploring different training methods to evaluate long‐term training effects; (3) include female athletes and participants with diverse training backgrounds; and (4) establish a connection between acute “optimal” power loads and long‐term adaptation outcomes.

## 5 CONCLUSIONS

This meta‐analysis synthesized data from 14 studies involving 197 participants to evaluate peak power during the PC and HPC across relative load ranges. Peak power increased substantially when loads increased from ≤40% 1RM to above 40% 1RM, whereas differences among the higher loading zones were generally small. This meta‐analysis indicates that loads above 40% 1RM generally elicit greater acute peak power than loads ≤40% 1RM during the PC and HPC. However, differences among the >40% to ≤70%, >70% to ≤85%, and >85% 1RM loading zones were generally small and statistically nonsignificant. Accordingly, the present findings are more consistent with a broad high‐power loading range above 40% 1RM than with a single statistically superior “optimal” load zone.

Under the acute testing conditions examined in the included studies, loads ≤40% 1RM generally produced lower peak power than loads above 40% 1RM. Loads within the >40% to ≤70% 1RM range can elicit relatively high acute peak power and may offer practical advantages when movement quality and fatigue management are important considerations. For acute peak‐power assessment or power‐oriented load selection, >70% to ≤85% 1RM may be considered one practical option within this broader range; however, this recommendation should not be interpreted as evidence that >70% to ≤85% 1RM produces greater peak power than either >40% to ≤70% 1RM or >85% 1RM.

Finally, the >70% to ≤85% 1RM range considered for acute peak‐power expression remains relatively broad. Further research is needed to refine this range across different populations and to determine whether the loads that maximize acute peak power also optimize long‐term training adaptations. These recommendations should primarily inform acute peak‐power assessment and programming decisions based on immediate power responses. They should not be interpreted as evidence that the same loading ranges are optimal for long‐term improvements in strength, power, or sport‐specific performance. Optimal loading for chronic adaptations remains uncertain and requires confirmation in longitudinal training studies.

## AUTHOR CONTRIBUTIONS

**Jiaxuan Sun:** Conceptualization; data curation; formal analysis. **Yiming Wu:** Data curation; formal analysis. **Yifan Zhang:** Formal analysis. **Li Li:** Supervision. **Lijun Shi:** Supervision.

## FUNDING INFORMATION

This meta‐analysis was funded by the Special Fund for Basic Scientific Research Business Expenses of Central Universities (Grant Nos. 2026KYPT05 and 2026KYPT06). The Laboratory of Sports Stress and Adaptation of General Administration of Sport provided only open‐access publication funds. The funders had no role in the study design, data collection, management, analysis, or interpretation; manuscript writing or review; or the decision to submit the manuscript for publication. At registration, this study was listed as a self‐funded project. After registration, access to open access funding for 2026KYPT05 and 2026KYPT06 was obtained.

## CONFLICT OF INTEREST STATEMENT

The author(s) have no conflicts of interest relevant to this article. The remaining authors declare that the research was conducted in the absence of any commercial or financial relationships that could be construed as a potential conflict of interest.

## ETHICS STATEMENT

The authors have nothing to report.

## SYSTEMATIC REVIEW PROTOCOL REGISTRATION

The protocol was registered in the PROSPERO database (#CRD420261296925).

## Supporting information


**Supporting Information S1.** PRISMA 2020 Main Checklist.

**Supporting Information S2.** The full search strategies for various databases.

**Supporting Information S3.** Domain‐level methodological quality assessment of the included studies.

**Supporting Information S4.** An overview of all studies.

**Supporting Information S5.** Funnel plot of the power clean and hang power clean.

## Data Availability

All data relevant to the study are included in the article or uploaded as Supporting Information.
