# Supplementary material for: Optimal power load ranges for the power clean and hang power clean weight‐lifting derivatives: A systematic review and meta‐analysis
Source: Physiol Rep. 2026 Nov 1;14(21):e71154. doi: 10.14814/phy2.71154 (PMC13725074; doi:10.14814/phy2.71154)

Funnel Plot of the power clean and hang power clean

(A: HPC; B: PC)

A1: Zone 1 vs. Zone 2

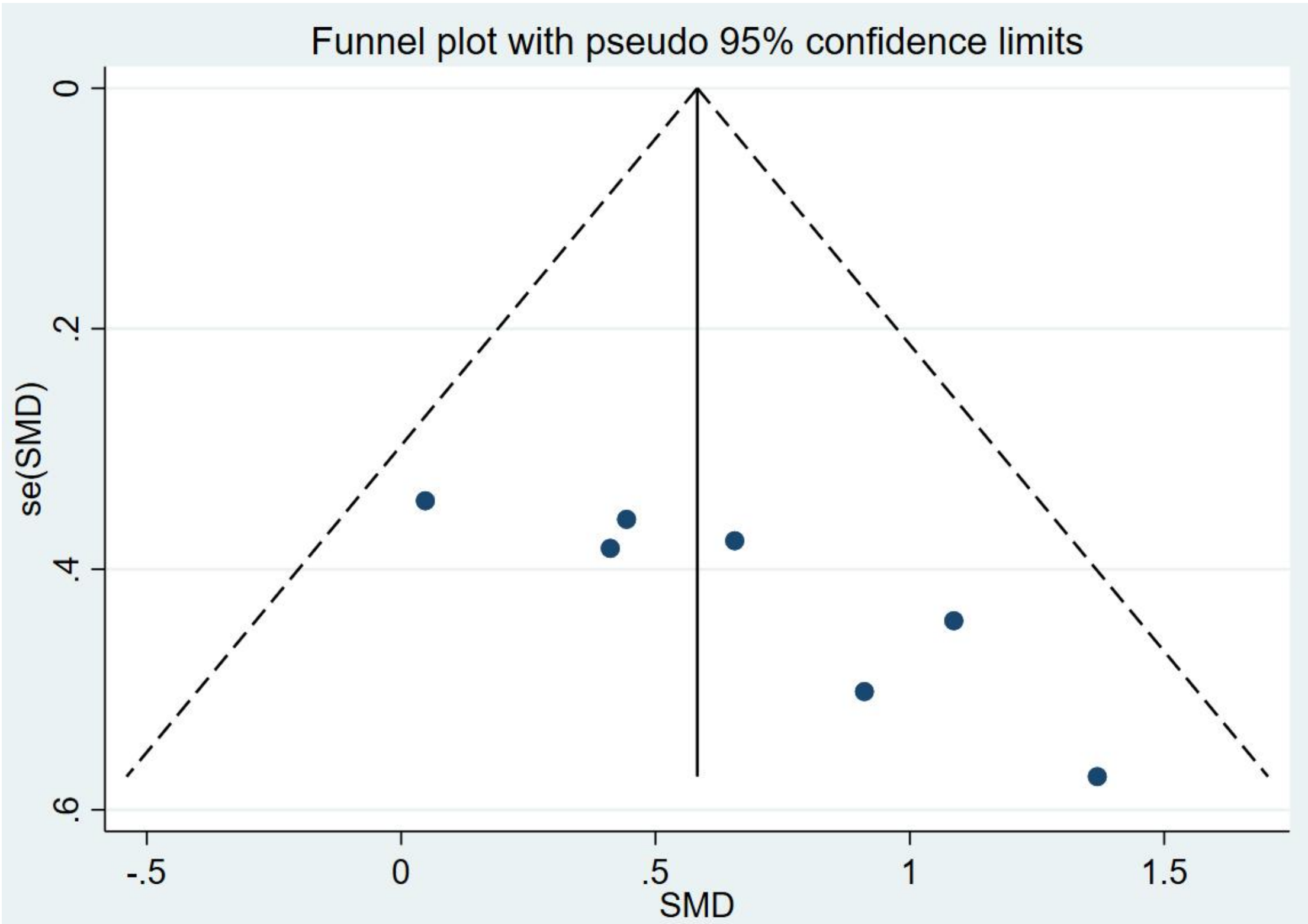

A2: Zone 1 vs. Zone 3

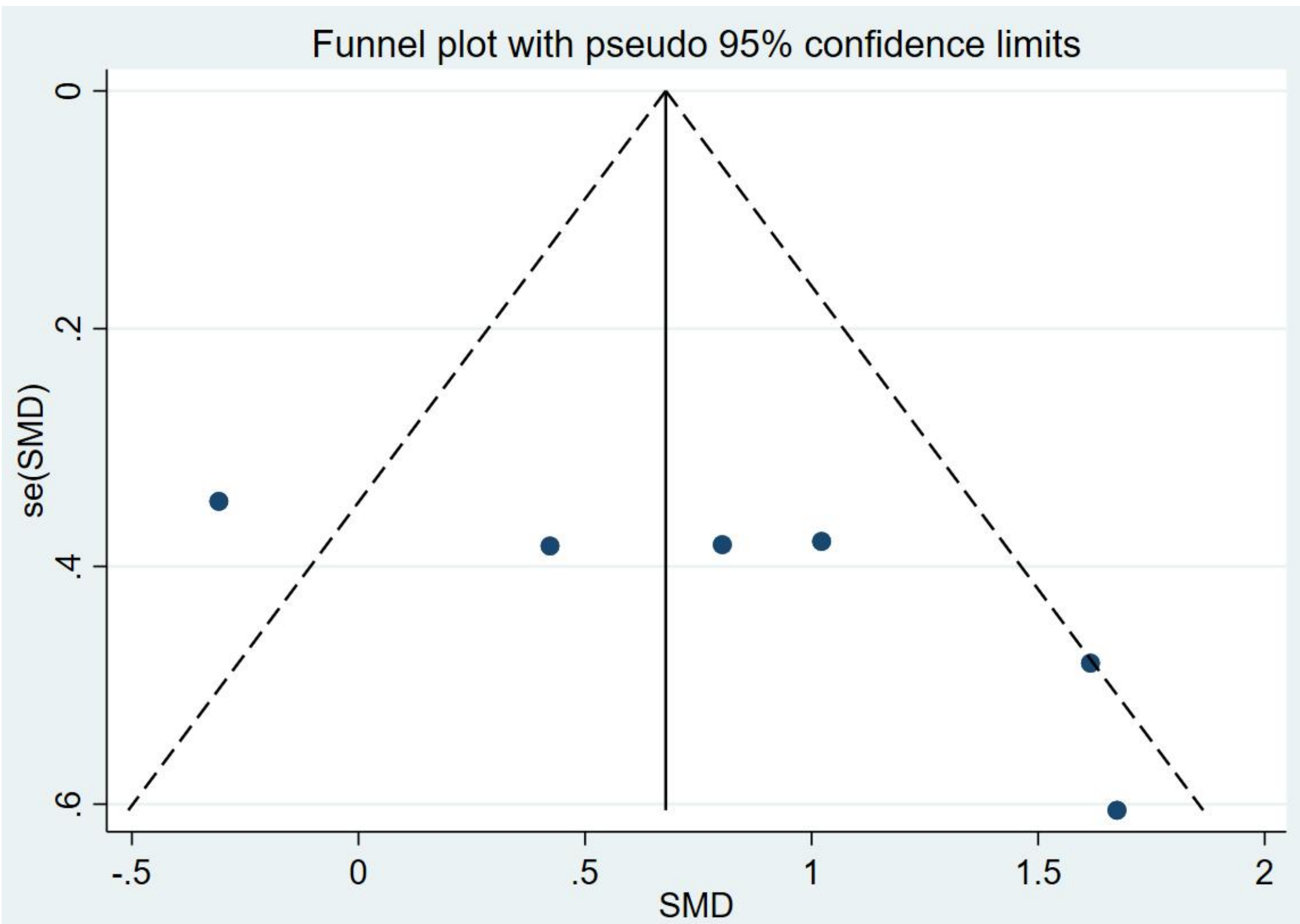

A3: Zone 1 vs. Zone 4

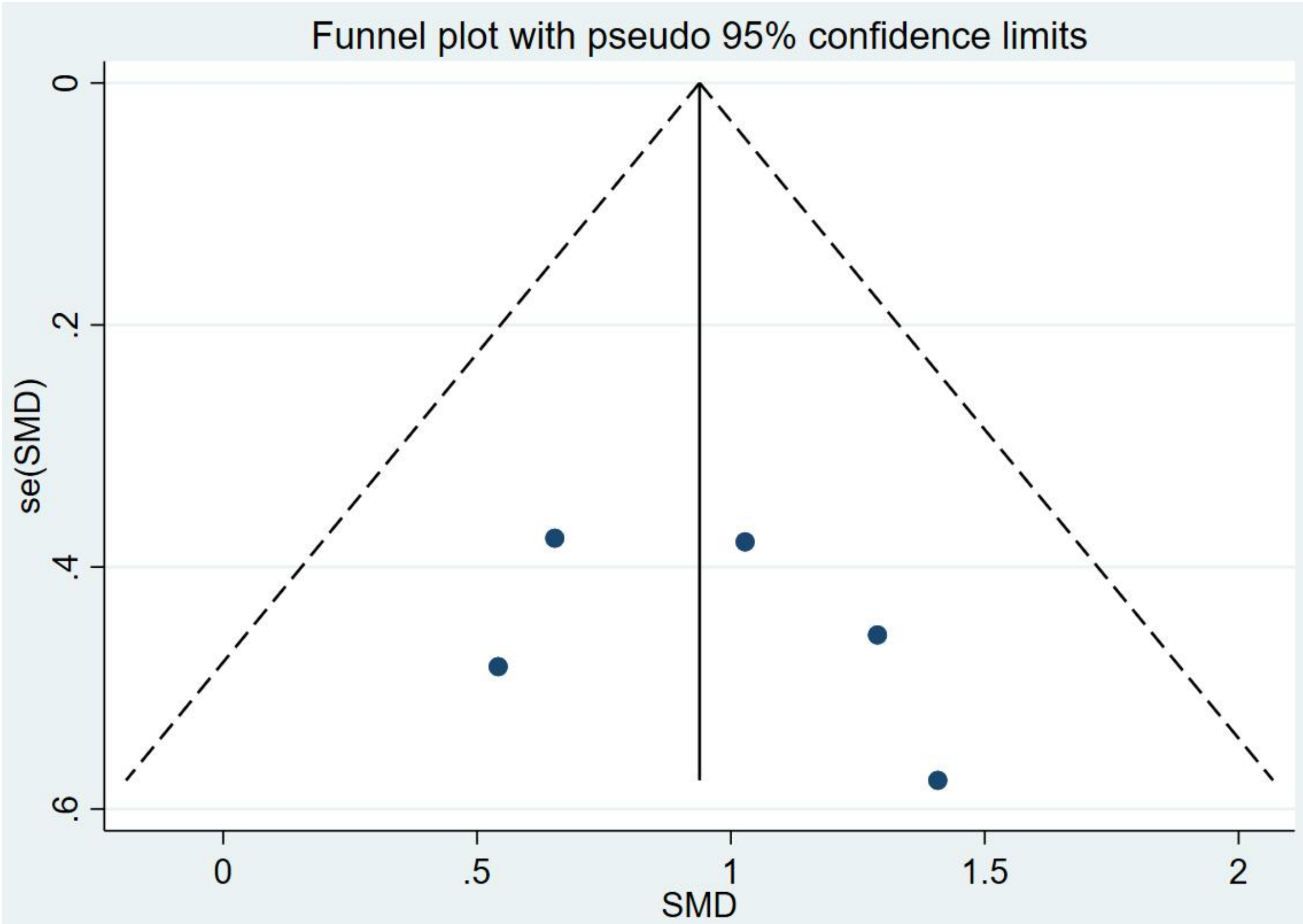

A4: Zone 2 vs. Zone 3

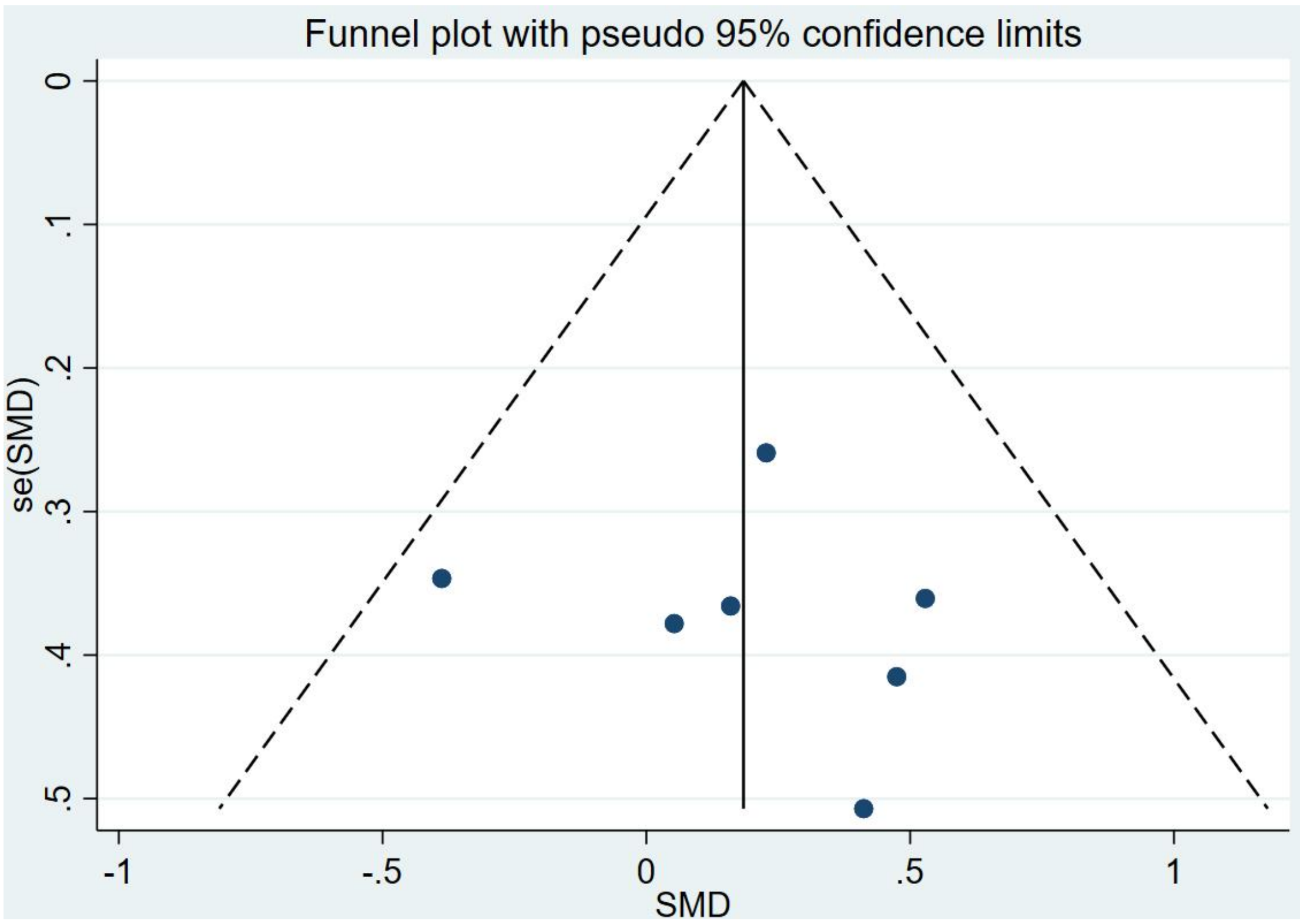

A5: Zone 2 vs. Zone 4

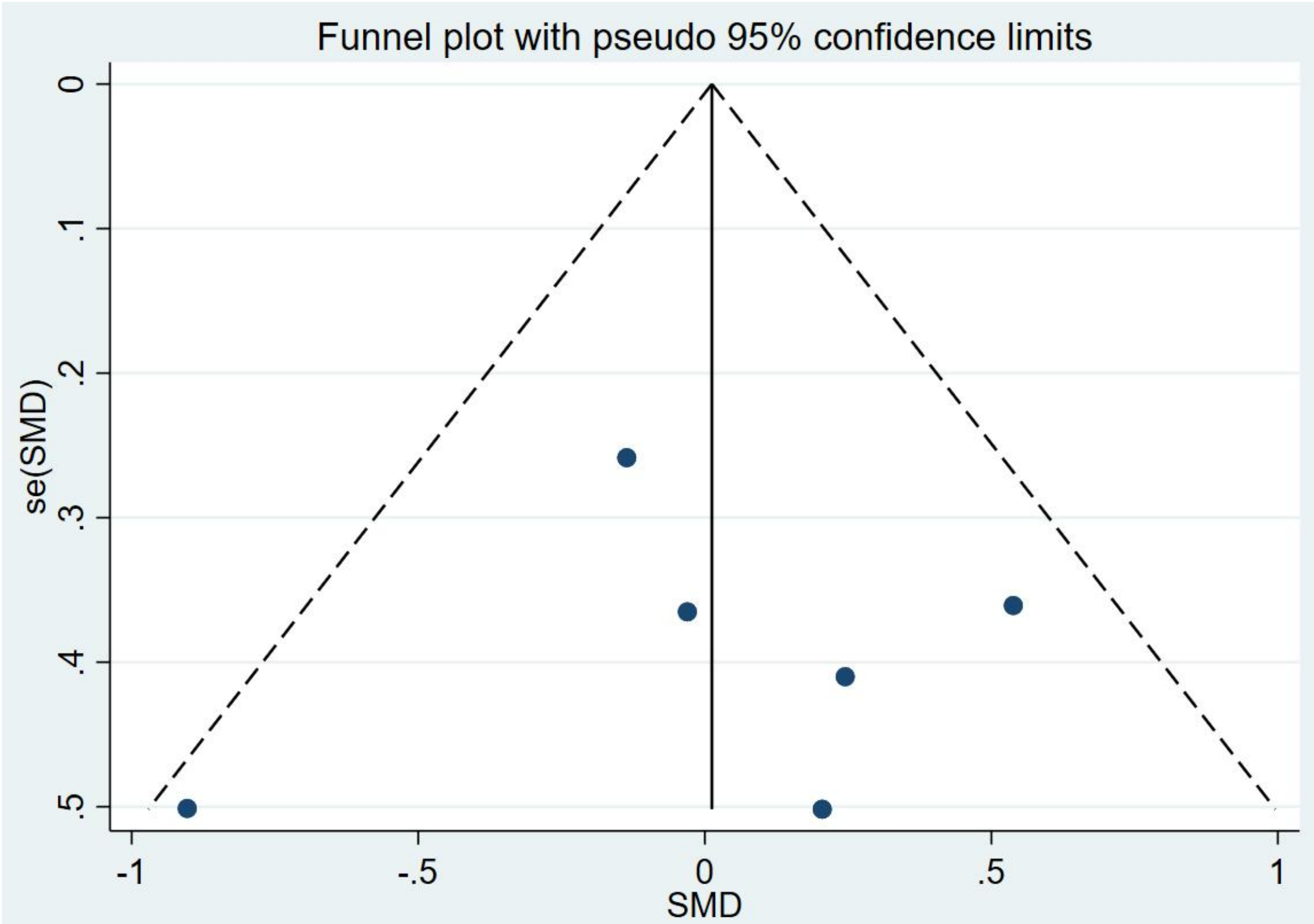

A6: Zone 3 vs. Zone 4

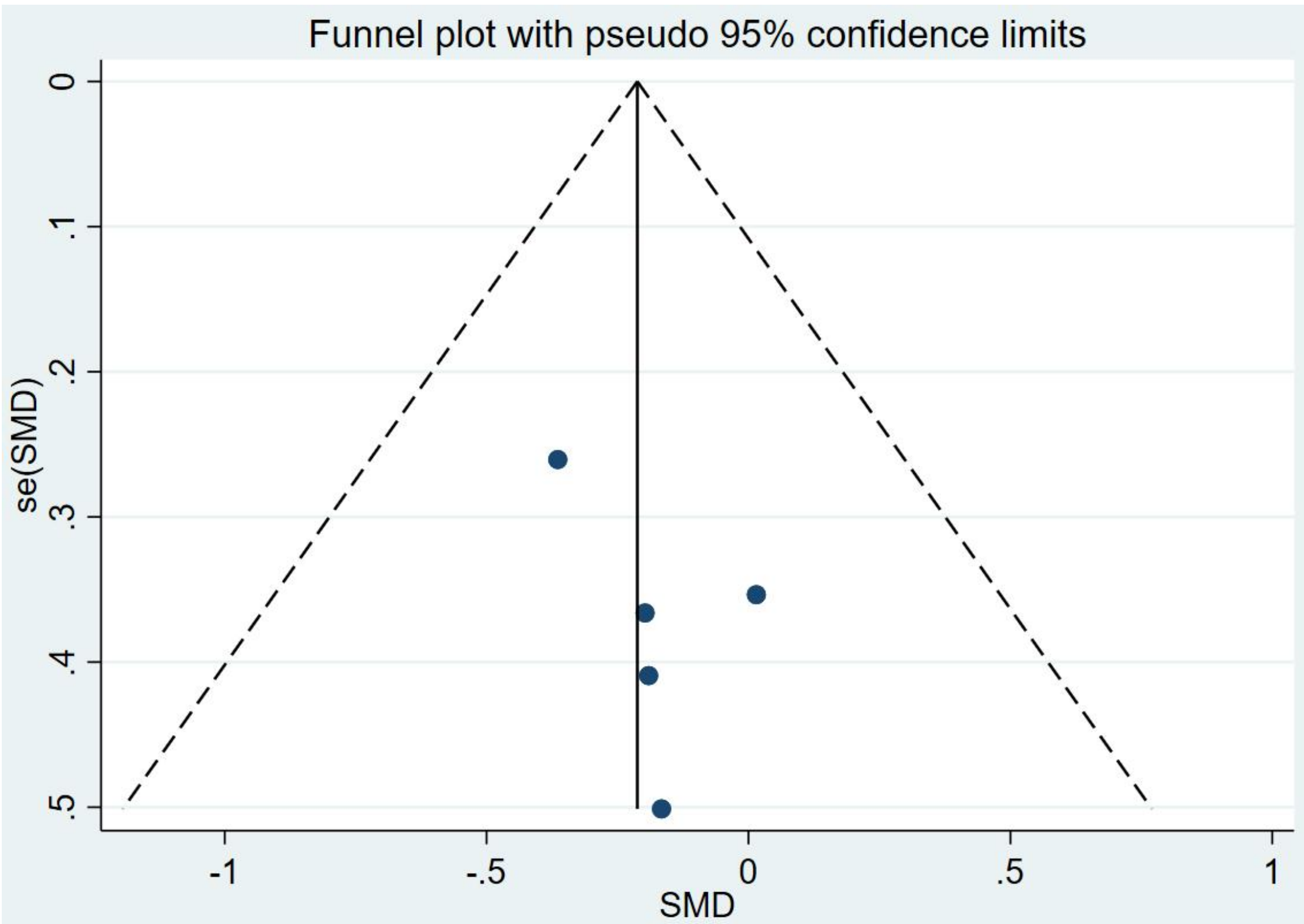

B1: Zone 1 vs. Zone 2

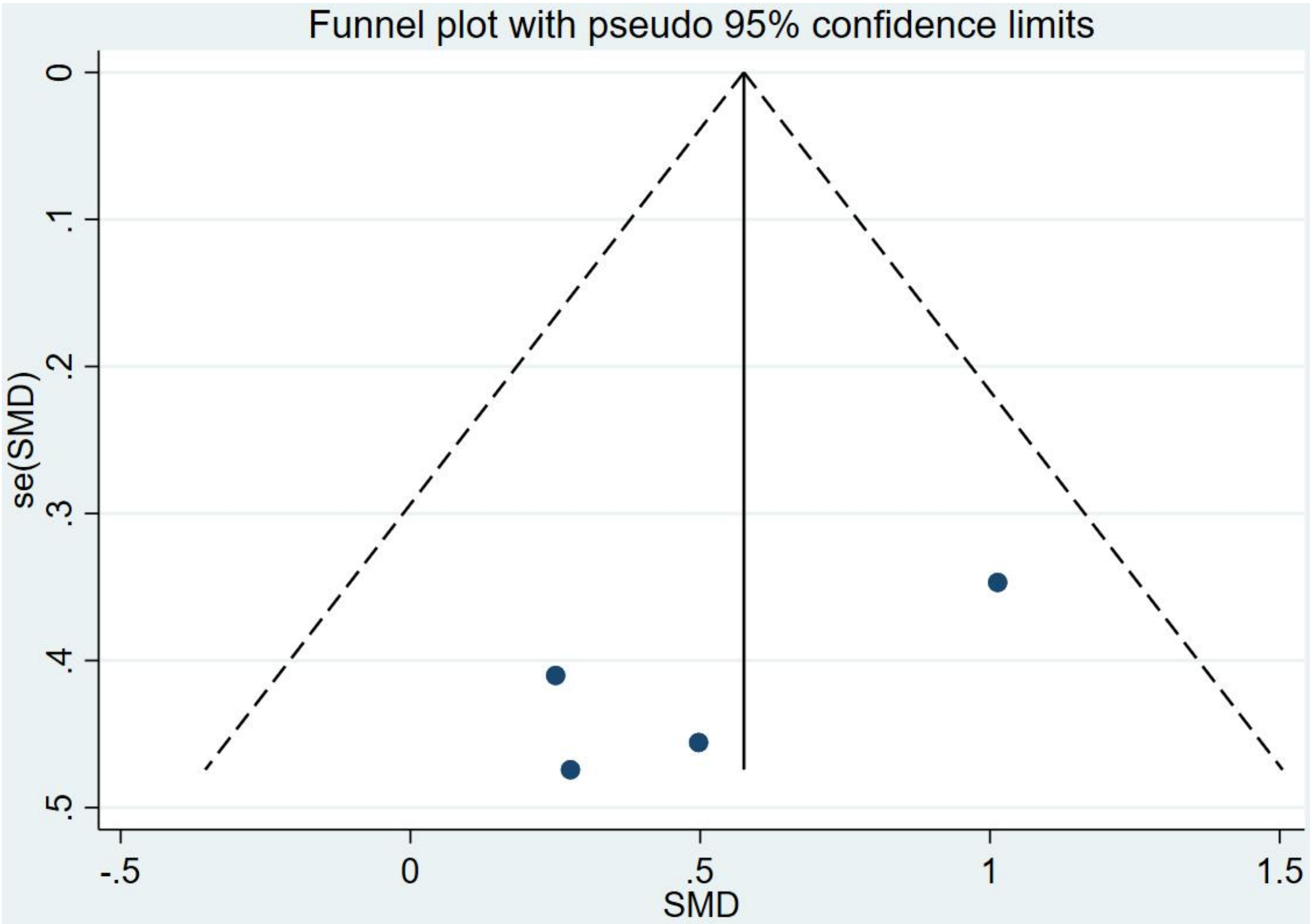

B2: Zone 1 vs. Zone 3

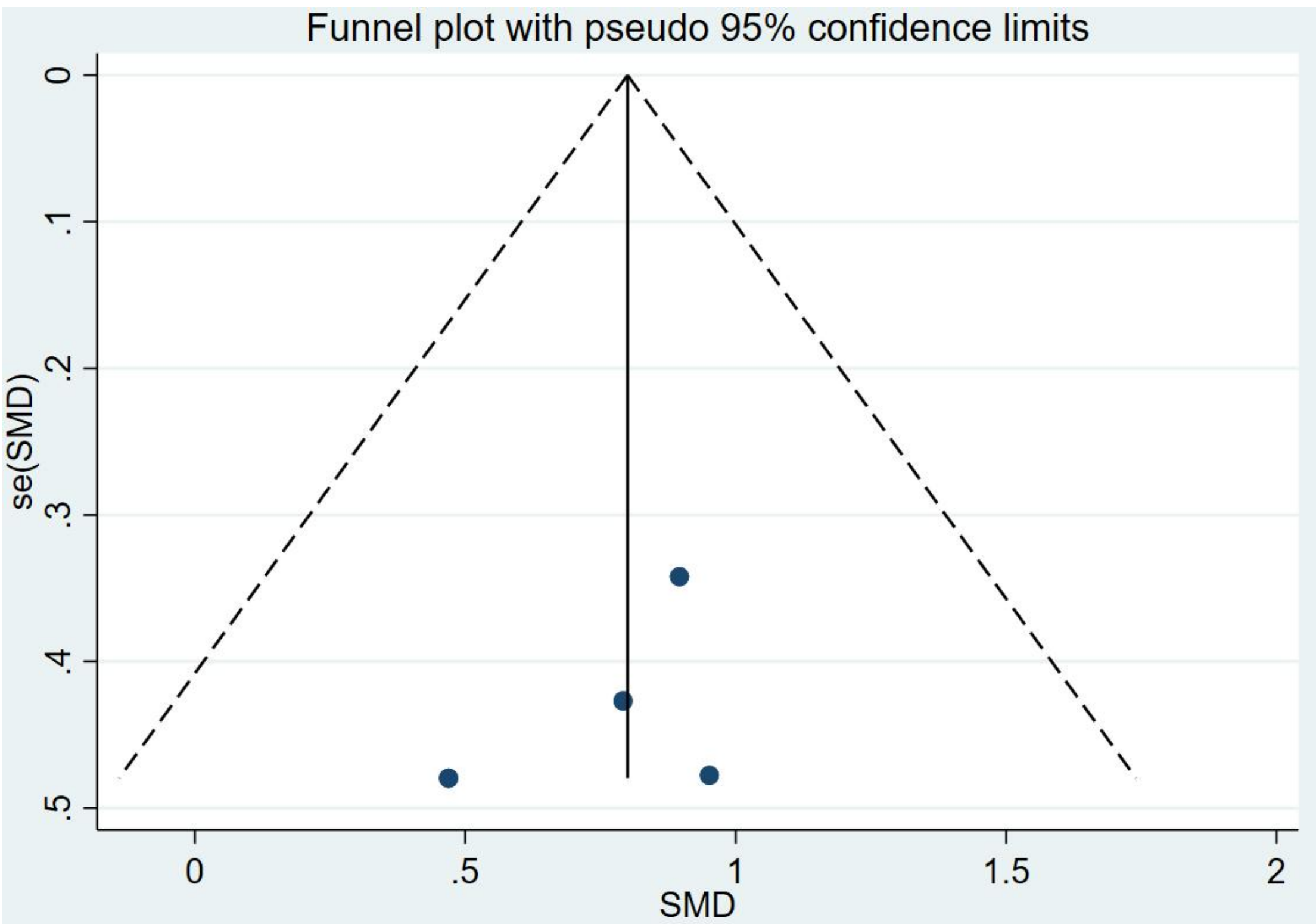

B3: Zone 1 vs. Zone 4

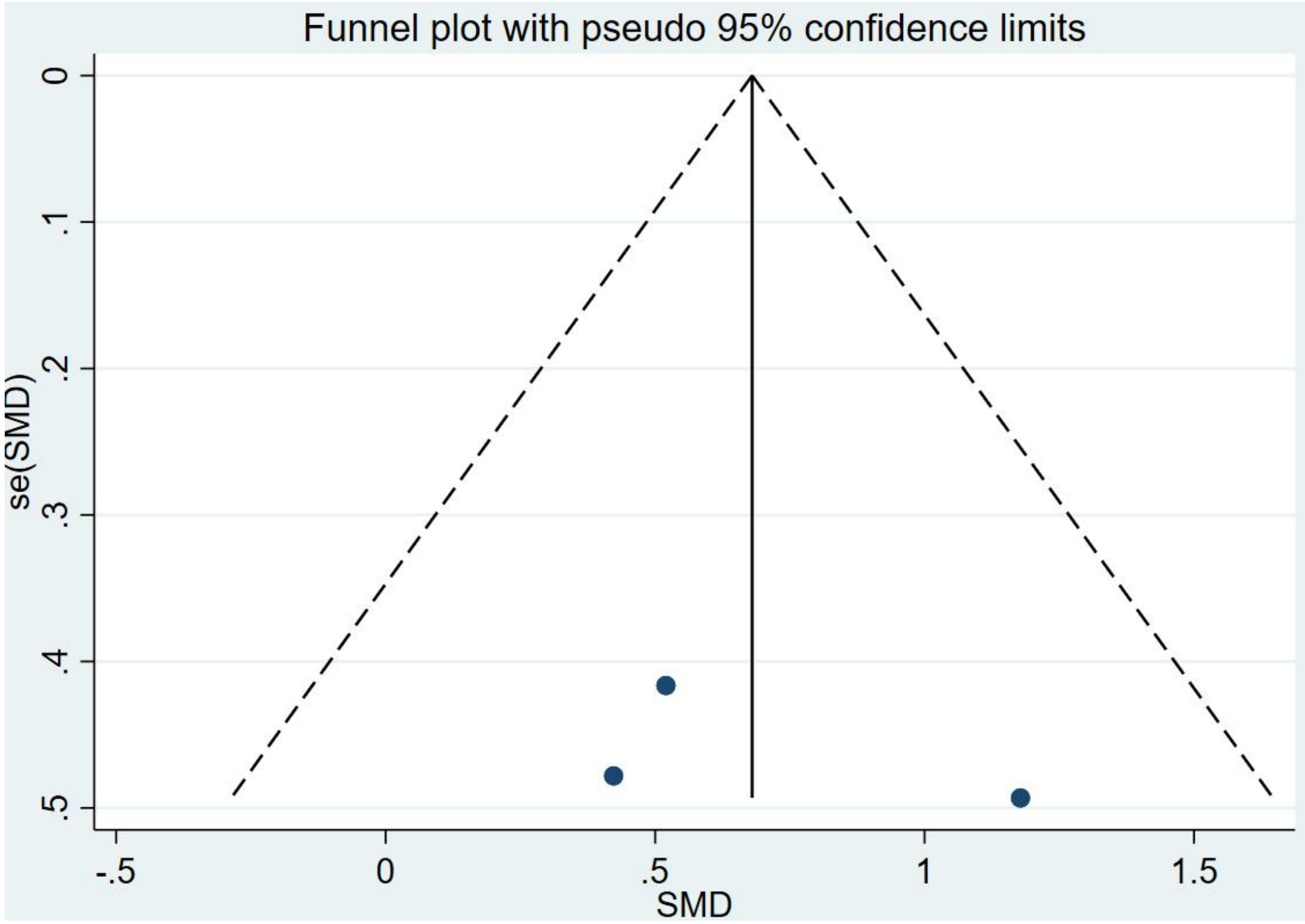

B4: Zone 2 vs. Zone 3

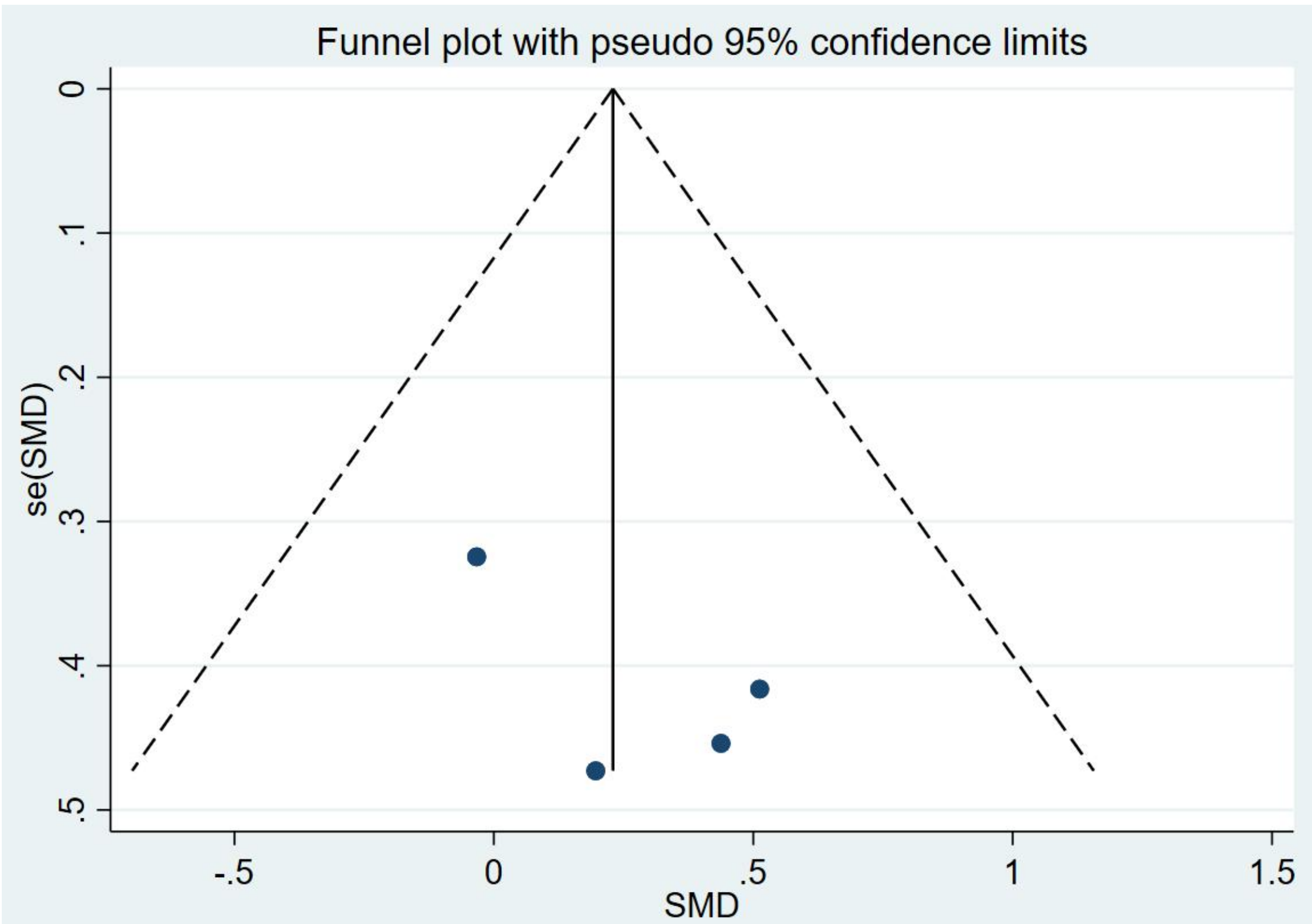

B5: Zone 2 vs. Zone 4

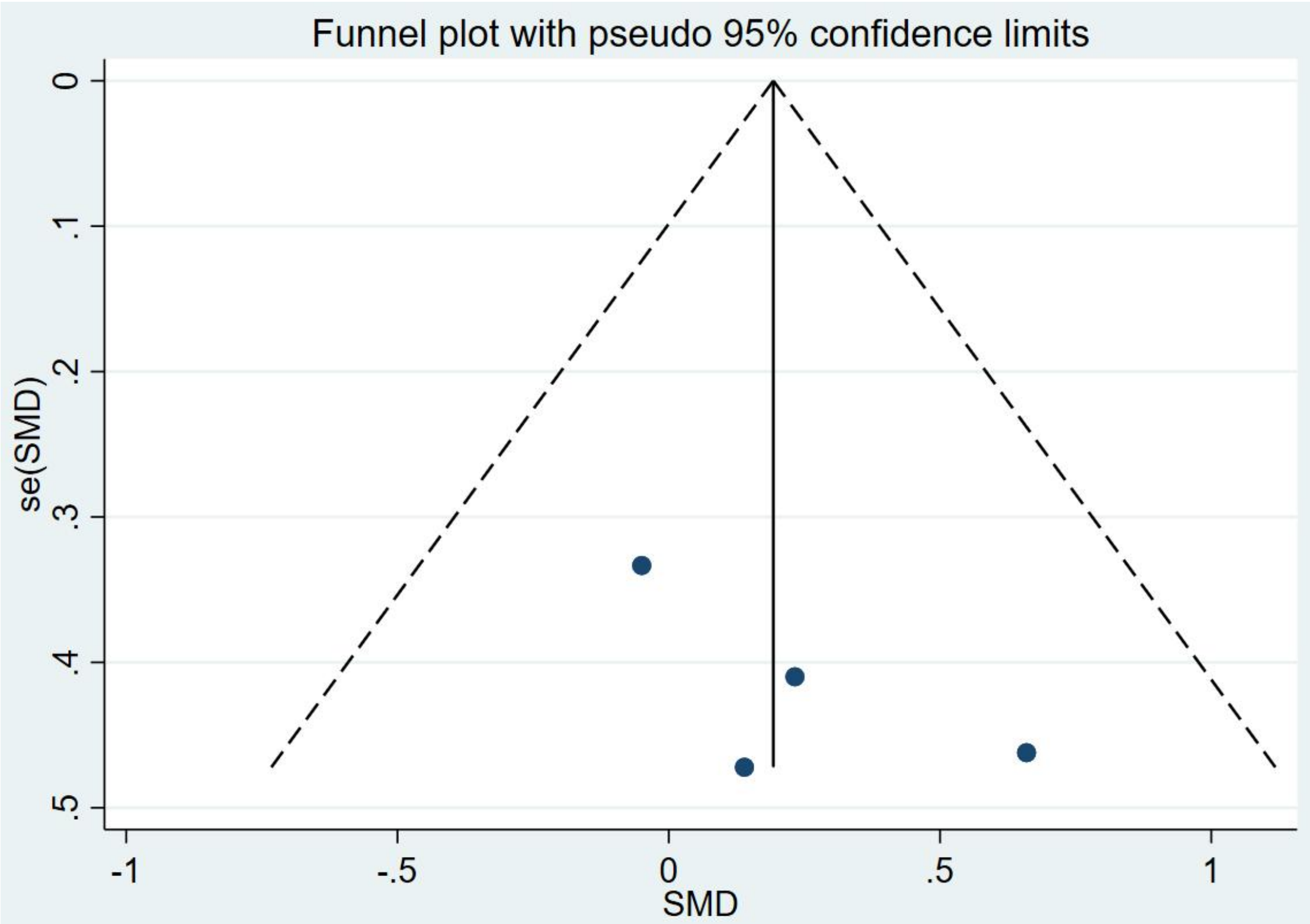

B6: Zone 3 vs. Zone 4

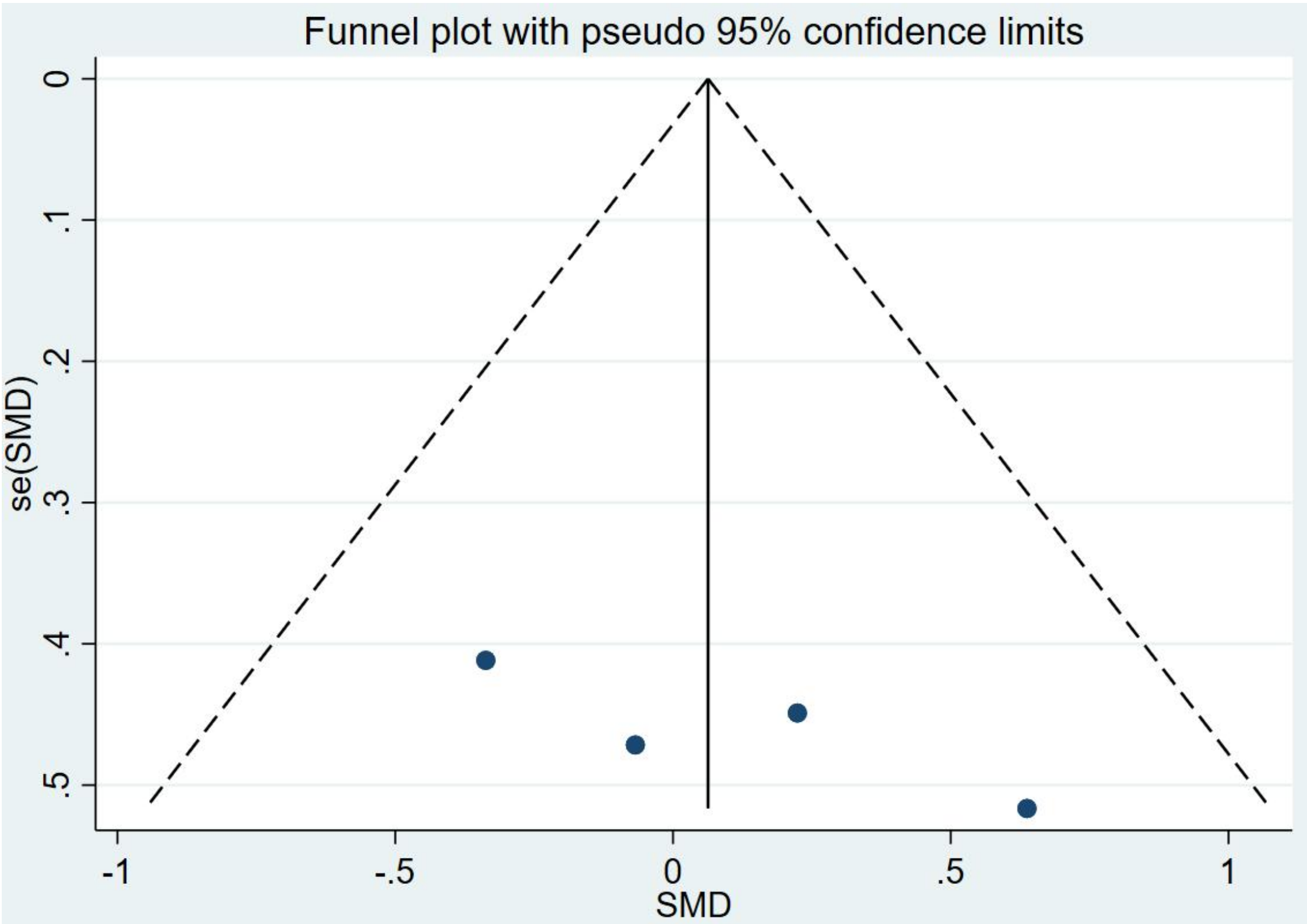

Supplement: Supplementary file 1 [file PHY2-14-e71154-s003.pdf]
