# Supplementary material for: Optimal power load ranges for the power clean and hang power clean weight‐lifting derivatives: A systematic review and meta‐analysis
Source: Physiol Rep. 2026 Nov 1;14(21):e71154. doi: 10.14814/phy2.71154 (PMC13725074; doi:10.14814/phy2.71154)
Supplement: Supplementary file 2 [file PHY2-14-e71154-s004.pdf]

The full search strategies for various databases

PubMed	((("power clean") OR ("hang power clean")) OR ("power training")) AND (((((("mechanical power") OR ("maximum power")) OR ("power production")) OR ("power development")) OR ("peak power")) OR ("optimal load")) OR ("power－load curve"))
Web of Science	#1 “power clean” (All Fields) or “hang power clean” (All Fields) or “power training” (All Fields)<br>#2 “mechanical power” (All Fields) or “maximum power” (All Fields) or “power production” (All Fields) or “power development” (All Fields) or “peak power” (All Fields) or “optimal load” (All Fields) or “power－load curve” (All Fields)<br>#3 #1 and #2
EBSCO	S1 “power clean” OR “hang power clean” OR “power training”<br>S2 “mechanical power” OR “maximum power” OR “power production” OR “power development” OR “peak power” OR “optimal load” OR “power–load curve”<br>S3 S1 and S2
EMBASE	#1 'power clean' OR 'hang power clean' OR 'power training'/exp OR 'power training'<br>#2 'mechanical power'/exp OR 'mechanical power' OR 'maximum power' OR 'power production' OR 'power development' OR 'peak power'/exp OR 'peak power' OR 'optimal load' OR 'power－load curve'<br>#3 #1 and #2
