# Supplementary material for: Optimal power load ranges for the power clean and hang power clean weight‐lifting derivatives: A systematic review and meta‐analysis
Source: Physiol Rep. 2026 Nov 1;14(21):e71154. doi: 10.14814/phy2.71154 (PMC13725074; doi:10.14814/phy2.71154)
Supplement: Supplementary file 4 [file PHY2-14-e71154-s005.docx]

**S3 Domain-level methodological quality assessment of the included studies**

| **Included study** | **D1 Sample** | **D2 Procedure** | **D3 Intervention** | **D4 Measurement / data** | **D5 Results** | **Total /5** |
| --- | --- | --- | --- | --- | --- | --- |
| Xie et al., 2025 | 1 | 1 | 1 | 1 | 1 | 5 |
| Mehls et al., 2022 | 1 | 0 | 1 | 0 | 1 | 3 |
| Takei et al., 2021 | 1 | 0 | 1 | 1 | 1 | 4 |
| Hayashi et al., 2021 | 1 | 1 | 1 | 1 | 1 | 5 |
| Suchomel et al., 2014a | 1 | 1 | 1 | 1 | 1 | 5 |
| Suchomel et al., 2014b | 1 | 1 | 1 | 1 | 1 | 5 |
| Comfort et al., 2012 | 1 | 1 | 1 | 1 | 1 | 5 |
| McBride et al., 2011 | 1 | 1 | 1 | 1 | 1 | 5 |
| Pennington et al., 2010 | 0 | 0 | 0 | 0 | 1 | 1 |
| Kilduff et al., 2007 | 1 | 1 | 1 | 1 | 1 | 5 |
| Cormie et al., 2007a | 1 | 1 | 1 | 1 | 1 | 5 |
| Cormie et al., 2007b | 0 | 1 | 1 | 1 | 1 | 4 |
| Winchester et al., 2005 | 1 | 1 | 1 | 1 | 1 | 5 |
| Kawamori et al., 2005 | 1 | 1 | 1 | 1 | 1 | 5 |

A score of 1 indicates that the corresponding methodological criterion was adequately met or reported, whereas a score of 0 indicates that the criterion was not met or that insufficient information was available. The summed score ranges from 0 to 5 and is provided only as a descriptive summary of methodological quality. These scores were not used for study exclusion or statistical weighting in the meta-analysis.
